# Factors associated with moderate wasting among marginalized 6 to 23-month aged children in Bangladesh: Findings of the Suchana program baseline survey data

**DOI:** 10.1371/journal.pone.0236786

**Published:** 2020-08-20

**Authors:** Mohammad Jyoti Raihan, Nuzhat Choudhury, Md Ahshanul Haque, Fahmida Dil Farzana, Mohammad Ali, S. M. Tanvir Ahmed, Sheikh Shahed Rahman, A. S. G. Faruque, Tahmeed Ahmed

**Affiliations:** 1 Nutrition and Clinical Services Division, Mohakhali, Dhaka, Bangladesh; 2 Department of Anthropology, Durham University, Durham, United Kingdom; 3 Save the Children Bangladesh, Dhaka, Bangladesh; African Population and Health Research Center, KENYA

## Abstract

Suchana—a large-scale, 7-year nutrition program that started in 2015—is being implemented in 250,000 households in the marginalized segment in north-east Bangladesh, with the aim of improving childhood nutrition status. Untreated childhood moderate wasting may develop to severe wasting, which is associated with a 10-fold higher risk of mortality compared to children of normal weight relative to height/length. Identifying the diverse, age-specific risk factors for moderate wasting may help such programs to formulate tailored interventions to prevent and treat childhood malnutrition in rural communities. The objective of this study was to identify the age-specific factors associated with moderate wasting among 6‒23-month-old children in beneficiary households. Cross-sectional data on 4,400 children was collected through systematic sampling between November 2016 and February 2017 using the Suchana beneficiary list. In total, 8.1% of 6‒11 month-olds and 10.3% of 12‒23 month-olds suffered moderate wasting; 12‒23-month-olds had a 1.3-fold higher risk of moderate wasting than 6‒11-month-olds. Our results of logistic regression models suggest that larger household size, higher maternal body mass index (BMI), and maternal food consumption status more than usual during the recent pregnancy were associated with a reduced risk of moderate wasting among 6‒11-month-olds. Higher maternal BMI, normal maternal food consumption status during last pregnancy, being female and maternal knowledge on diarrheal management, were associated with a reduced risk of moderate wasting among 12‒23-month-olds. In conclusion, beyond maternal BMI and maternal food consumption status during the last pregnancy, the factors associated with moderate wasting among 6‒23-month-olds in the poorest households in Bangladesh are age-specific.

## Introduction

Globally, around 238 million children under five years-of-age are either undernourished or overweight [1]. Undernutrition contributes to around 35% of the total disease burden and half of all preventable deaths in children under five worldwide each year [2–4]. Around 49.5 million children under five globally suffer wasting [1]. Wasting occurs due to a deficiency in one or more macronutrients due to poor food intake, malabsorption or chronic inflammation [5]. Wasting is particularly pronounced in developing nations, with children in south and central Asia disproportionately affected [1, 2, 5–7] and is a major ongoing public health hazard that demands immediate attention [1, 8].

Acute malnutrition is characterized by excess loss of body weight for height/length (wasting), low mid-upper arm or nutritional edema [7]. Acute malnutrition is usually defined as low WHZ, low MUAC or nutritional edema among children between 6 months and 5 years [1, 9, 10]. Malnutrition can also be associated with other clinical conditions include—but are not limited to—fever, respiratory distress, heart failure, electrolyte imbalance, anorexia, anemia, profuse diarrhea, and even shock [11]. Wasting is associated with a three-fold higher risk of death from infectious diseases [3] and is responsible for around 14.6% of all mortalities attributed to nutritional measures among children under five [3].

In Southern Asia alone, if both WHZ-score and MUAC cut-offs are considered, 25.3 million under-fives suffer wasting, of whom 64% have moderate wasting [1] and 8.7 million are adversely affected by the more profound form of wasting called severe wasting [1]. Children with SAM have a 10-fold higher risk of death than healthy children [3, 12] and a mortality rate of 5–60% [7]. Increased susceptibility to infections further increases the odds of mortality for immunocompromised acutely malnourished children, thus the vicious cycle of infection-undernutrition continues if not treated [2].

The recent Demographic and Health Survey in Bangladesh revealed that around 14% children under-five were wasted, with the prevalence reaching 20% for 9‒11-month-olds [13], indicating that the risk of moderate wasting could be associated with age. Bangladesh achieved a sharp reduction in the prevalence of chronic child undernutrition/stunting of 15% between 2004 and 2014; however, the prevalence of wasting remained around 16% [13]. Bangladesh is still far from achieving the global target of reducing and maintaining childhood wasting below 5% [8]. In addition to treating children with SAM, it is imperative to treat children with moderate wasting—especially in low-and-middle income countries with a high burden of undernutrition—to achieve a global decline in child undernutrition. Children with moderate wasting are considered a vulnerable population; if immediate interventions are not undertaken to improve their nutrition status, children with moderate wasting may develop SAM, which is associated with increased mortality and morbidity [12].

Promoting and supporting breastfeeding, counseling on complementary feeding practices, energy-and nutrient-dense food supplementation and improving dietary diversity and quantity for older children are recommended to both prevention and treatment of MAM in the community [8, 14, 15]. These strategies do not require in-patient treatment and could potentially prevent around 54,000 deaths among children under five globally [15]. The degree of wasting and clinical complications in children with MAM are not the same to that of SAM children though the underlying causes or risk factors are similar [16]. Previous studies in Bangladesh using nationally representative data identified maternal body mass index (BMI), maternal exposure to media, maternal age, birth size of children, birth parity, history of morbidities, household wealth, paternal education and occupation status were significantly associated with both SAM and MAM in children under five [17–20].

Suchana is a large-scale, 7-year nutrition program serving around 250,000 poor households with the highest vulnerability to malnutrition in north-east Bangladesh; the program began in 2015 and will reach around 1.7 million people in the Sylhet and Moulvibazar districts [21]. Sylhet division was selected as it has the highest proportion of people living below the lower poverty line and has the poorest scores for several critical health and nutrition indicators [22, 23]. The primary aim of the program is to reduce the incidence of chronic undernutrition/stunting among children using a life cycle approach. The Suchana program targets the first 1000 days of life as a critical period of growth and development, in an attempt to provide long lasting beneficial effects throughout life [21]. The program activities focus heavily on identification and mitigation of the factors that affect child nutrition status in order to formulate scalable and replicable interventions to reduce the burden of child undernutrition among the poorest segment of society. The strict inclusion criteria ensured that only the poor and poorest segment of the population was enrolled.

The aim of this study is to identify the age-specific factors associated with the occurrence of moderate wasting among Suchana beneficiary children. In this study we have defined moderate wasting as the WHO cut-off of a WLZ-score of between– 2 to -3 and did not include cases as per MUAC cut-off values or observation of nutritional edema. This information may help the Suchana program and other large-scale nutrition programs with similar priorities to effectively divert and mobilize the necessary resources towards the root causes or domains that influence moderate wasting and inform the development of effective strategies to prevent malnutrition among children in poor households.

## Methods

This study was approved by the Research Review Committee and Ethical Review Committee, the two obligatory components of the institutional review board of International Centre for Diarrhoeal Disease Research, Bangladesh (icddr,b) under research protocol number PR-16020. Informed written consent was obtained from the parents of all children. The respondents were also informed that their participation was voluntary and of their right to withdraw themselves from the study at any point for any reason. Information that could reveal any respondent’s identity was not used while analyzing the data. The Suchana evaluation study has been registered at the Registry for International Development Impact Evaluations (RIDIE) as RIDIE-STUDY-ID-5d5678361809b.

### Study design, sample size and sampling

Cross-sectional data on 4,400 children aged 6‒23 months, including 1,200 children aged 6‒11 months and 3,200 aged 12‒23 months, was collected through the Suchana baseline survey using a structured questionnaire and anthropometric measurements.

The minimum sample size required to achieve 80% power and a 5% level of significance was determined using the proportion of 6‒11-month-olds in the Sylhet region who consumed four or more food groups (minimum acceptable diet) in the 24 hours preceding the survey (unpublished study in Sylhet, Bangladesh). The proportion of MAD used for calculation was 24.6% with an intra-cluster correlation (ICC) of 0.0395 (unpublished study in Sylhet, Bangladesh).

The minimum sample size required for the 12‒23-month-old group was based on the prevalence of stunting from the same unpublished study and an assumed difference of 6% between the intervention and control groups after three years of the intervention: we expected the prevalence of stunting in the control group to be around 47% at the end of the first 3 years of the Suchana program (assuming a decrease of 1% every year due to secular trends and an additional 2% per year due to the Suchana program). The minimum sample size required to detect a 6% difference in the prevalence of stunting between groups was calculated assuming 80% power, 5% significance and an ICC of 0.01 (published study in Sylhet, Bangladesh). The sample size calculations were conducted in Stata software (STATA Corp.) using the *clustersampsi* module.

The Suchana baseline survey was conducted between November 2016 and February 2017 in 80 unions (the smallest administrative unit in Bangladesh) of the Sylhet and Moulvibazar districts. Eighty unions (containing 640 villages) were selected from the unions in the two districts using a lottery method. Vulnerable villages in each of the selected unions were identified through consultation with local elites, such as local teachers, local administrators, religious leaders, local non-government and government authorities, and local political leaders. Depending on the population of each village, one or two wealth ranking sessions were conducted in each village following a participatory rural appraisal strategy [24], in which the villagers identified marginalized households in their villages. The Suchana beneficiary inclusion questionnaire was maintained to verify the identified marginalized households.

Households in which (i) the members of the household could not have three full meals a day throughout the year; (ii) the monthly household income was less than Tk. 7500 (1 USD = ~ Tk. 82); (iii) with total household business assets worth less than Tk. 15,000 (excluding land, ponds or dykes); (iv) with less than 10 decimals of homestead land or (v) with less than 50 decimals of cultivable land (excluding land, ponds or dykes) were eligible for enrollment if any one of the following conditions was satisfied: (i) the household has a married women between 15 to 45-years-old; (ii) the household has a pregnant woman (including divorcees and widows); (iii) the household has at least one child under two-years-old or at least one female adolescent aged between 15 and 19-years-old. However, households in which the beneficiary was pregnant were excluded from the sampling list for the evaluation as reported data on estimated maternal body mass index (BMI) would be inaccurate or overestimated. Households were selected for the baseline survey using a systematic sampling strategy by calculating an ‘interval’ using the total number of eligible potential beneficiary households available and the minimum sample size required. The calculated interval for selecting households was 1.89, with the first household chosen randomly from the household list. Informed written consent was obtained from the mothers of the children after the purpose of the survey was clearly explained to them in Bengali language and a copy of the consent form was provided to the participants for future reference.

### Data collection

Data were collected using Personal Digital Assistants (PDAs) by field staff members after completion of three months’ training on administering the questionnaire and anthropometry (Please see S1 File). Selected data collectors with special training on anthropometry were assigned to collect anthropometry data to minimize measurement error and inter-individual measurement variability. Body weight was recorded using SECA 874 model weight scales to an accuracy of 0.1 kg or 100 gm. The mothers were weighed wearing minimum culturally acceptable clothing with no added ornaments, headscarf or other garments that may lead to an overestimation of weight. Then, the mothers were weighed holding their youngest/index child wearing minimal clothing. The PDAs automatically calculated the child’s weight.

Children’s length was measured using SECA 416 infantometers to 0.1 cm precision and maternal height was measured using wooden scales similar in design to those used in the Bangladesh National Micronutrient Survey and other government projects. A third reading was taken if the difference between the first two consecutive readings for the children’s length and mother’s height were more than 0.1 cm and 2 cm, respectively, and the values were averaged during data analysis.

### Data management

An independent quality control (QC) team randomly re-visited 5% of the surveyed households and re-examined 25 key indicators from the original survey. Immediate feedback was provided to the survey team if any discrepancies were found. During the survey, all data collectors checked the collected data on-site and uploaded the data after the responses had been scrutinized by field supervisors.

The data management team downloaded the data from the server, performed range, duplication, consistency and frequency checks and conducted cross-tabulation. If any inconsistencies were found, the field supervisors were immediately informed and took rectifying steps. The downloaded data was stored in a password-protected personal computer in a specific drive or folder that could only be accessed by selected investigators. After a specific time period, all data was backed up on three storage instruments: another drive and folder on the same computer, the principal investigator’s computer, and an external password-protected hard drive placed in both secure location and a secure building.

### Variables

The outcome variable was the occurrence of moderate wasting, as defined by the WHO as a cut-off of weight-for-length z-score (WLZ) between -3 and -2. A WLZ-score below -3 was considered severe wasting [25]. The variables used during analyses were broadly categorized into six domains, namely socio-economic status, general maternal characteristics, maternal reproductive characteristics, maternal knowledge, child characteristics, and child feeding practices. Some variables used in this study were already included as such in the questionnaire; others were computed using multiple variables from the questionnaire. All categorical variables are defined in Table 1; total household yearly income from all sources, household size, maternal age, maternal BMI, and the total number of maternal pregnancies were examined as continuous variables. The variables examined are relevant to wasting, as indicated by the literature [17–20, 26, 27] and the study protocol.

**Table 1:** Categorized variables and operational definition/notes.

Variable		Categorization/cut-off values	Operational definition/note
General child characteristics			
	Gender	‘Male/Female’	
	Birth order	1. 1	
		2. 2–3	
		3. 4–5	
		4. 6 or above	
	Morbidity status in last 15 days	‘Yes/No’	A child was declared to have morbidity if at least one of the following conditions was present during the 15 days preceding the survey: fever, pain/ache, weakness, cold/cough, skin rash, loose bowel movement/diarrhea, drowsiness, vomiting, lack of appetite, otitis/ hearing problem, dental problem, asthma, or throat swelling
		
Child feeding practices			
	Received colostrum	‘Yes/No’	
	Initiation of breastfeeding within 1 hour of birth	‘Yes/No’	
	Received Minimum Acceptable Diet (MAD)	‘Yes/No’	Composite score calculated according to the FANTA guideline [51]
Socio-economic status			
	Household size	Continuous measure	Number of persons living in the household. A household member was defined as someone who consumed meals from the same cooking pot in the six months preceding the survey
	Household yearly income	Continuous measure	Total income of all household members in the year preceding the survey
	Household savings by spouses	Continuous measure	Total savings made by the incumbent spouses in the year preceding the survey
	Asset index	Quintiles	Asset index was constructed by principal component analysis [13] using the variables: ownership of cows, chickens and ducks, birds e.g. pigeons, goats/sheep, fish, plough, unit for keeping livestock (cattle house), shop premises, unit for storing crops, boat, fish net, rickshaw/van, trees (above 100 Tk.), sewing machine, radio/cassette player/DVD/CD player, television, electric fan, mobile phone, bicycle, chair, table, *chouki* (cot), sofa (any type), mosquito net, ceremonial sarees, floor, roof and wall material of the house, as well as ownership of house(s) and number of rooms in the household, along with the type of fuel used for cooking
	Household Food Insecurity Access Scale	1. Food secure	HFIAS was used as a measure of household food security status. Households were categorized into four categories as per the Food and Nutrition Technical Assistance guidelines, which is a continuous measure of the degree of food insecurity (access component) in a household [52]
		2. Mildly food insecure	
		3. Moderately food insecure	
		4. Severely food insecure	
General maternal characteristics			
	Maternal age	Continuous measure	Calculated in ‘years’
	Maternal BMI	Continuous measure	Calculated using maternal weight and height (kg/m^2^)
	Maternal education status	‘No schooling/some schooling’	‘No schooling’ if no formal education or ‘Some schooling’ if at least 1 year of schooling from any formal education institution
	Maternal occupation	‘Earning’/’Non-earning’	Non-earning occupations are occupations which does not provide any money or financial remuneration e.g. being a housewife, full-time student or doing nothing to earn money
	Maternal handwashing practices during three critical times	‘Yes/No’	The critical times for handwashing are:
			1. Before eating
			2. Before preparing food
			3. Before feeding a child
			4. After defecation
			5. After cleaning a child’s bottom
Maternal reproductive characteristics			
	Iron and folic acid (IFA) supplementation status during last pregnancy	‘Yes/No’	‘Yes’ if any IFA tablets were consumed during the last pregnancy
	Place of ANC visits	‘Appropriate/Inappropriate’	‘Appropriate’ if mother visited: UpaZila/government medical health complex/Zila hospital/maternity center/family planning center/ RAC *shushastho* clinic/private hospital/clinic/private doctor chamber/local NGO health center/satellite clinic/EPI center
	ANC provider	‘Qualified/Unqualified’	‘Qualified’ if the provider was: a BRAC *shasthoshebika*/family welfare visitor (FWV)/female welfare assistant (FWA)/skilled birth attendant (SCBA)/doctor with MBBS degree/nurse/paramedic
	Attendant during last child delivery	‘Medically trained/Unqualified’	The delivery attendant was considered medically trained if the attendant was: a FWV/SBCA/nurse/paramedic/doctor with MBBS degree
	Type of delivery (normal)		Normal vaginal delivery
	Place of delivery (home)		Indexed child born in the house
	Received TT vaccination during pregnancy	‘Yes/No’	
	Total number of maternal pregnancies	Continuous measure	Total number of pregnancies for the mother including abortion/menstrual regulation and last pregnancy
	Maternal food consumption status or quantity of food consumed every day during the last pregnancy	1. Less than usual	
		2. Same as usual	
		3. More than usual	
	Maternal resting status during last pregnancy	1. Less than usual	
		2. Same as usual	
		3. More than usual	
Maternal knowledge			
	Maternal knowledge on the consumption of IFA supplementation during pregnancy	‘Yes/No’	‘Yes’, if the mother knew about the appropriateness of consuming IFA during pregnancy
	Maternal knowledge on at least one consequence of early pregnancy	‘Yes/No’	‘Yes’, if the mother mentioned at least one of the following occurrences: low birth weight baby/preterm birth/pregnancy-induced hypertension/anemia/urinary tract infection/abnormal labor or caesarean section for delivery
	Maternal knowledge on the minimum number of ANC visits	‘Correctly knows/Do not know’	Mother was declared to have correctly known the minimum number of ANC visits required if her response was 4 or any number above 4.
	Maternal knowledge on the appropriate institution(s) available for medical treatment in the community	‘Yes/No’	‘Yes’ if mother mentioned at least one of the following: health and family planning centre/UpaZila or government medical hospital/ medical college hospital/district hospital/ chamber of a doctor with MBBS degree/ community clinic
	Maternal knowledge on available medically trained healthcare provider(s)	‘Yes/No’	‘Yes’ if mother mentioned at least one of the following: BRAC shasthoshebika/FWV/FWA/SCBA/doctor with MBBS degree/nurse/paramedic
	Maternal knowledge on the initiation of breastfeeding	‘Yes/No’	‘Yes’, if her response was the initiation of breastfeeding within 1 hour of birth
	Maternal knowledge of exclusive breastfeeding	‘Yes/No’	‘Yes’, if her response was that the child should not consume anything except for breastmilk and medicines including oral rehydration salt (ORS) up to 6 months-of-age
	Maternal knowledge of diarrheal management	‘Yes/No’	‘Yes’, if mothers knew about treating children with oral rehydration salt and zinc supplementation during diarrhea

### Statistical analysis

All statistical tests were performed in Stata 15 software. The z-scores were calculated using the ZSCORE06 package of Stata 15 [28]. Descriptive statistics were produced using univariate analyses to describe variables. Proportions (%), frequencies and total numbers of children were reported for categorical variables; means, standard deviations (SD) or median, interquartile range (IQR) and sample sizes are shown for continuous variables. As data were collected from multiple unions/clusters or the smallest Government administrative unit of Bangladesh, the Stata’s *svyset* command was used to adjust for the clustering effect during bi-variate and multivariable analyses. Simple logistic regression and multiple logistic regression were used to illustrate the crude and independent relationships between the variables of interest and moderate wasting among the children by age groups (6–11 months and 12–23 months). *p*-values less than 0.05 were considered statistically significant.

All variables in the bi-variate analyses with a *p*-value < 0.05 were used in the conventional final multiple logistic regression models. Several post-estimation diagnostics such as collinearity diagnostics (*collin*), the Pearson or Hosmer-Lemeshow goodness-of-fit test (*lfit*), classification statistics and table (*lstat*) and the specification link test for single-equation models (*linktest*) were subsequently performed to test for multicollinearity between variables and assess the overall model fit and overall predictive accuracy of the models.

All variables in the final regression method were entered simultaneously according to the ‘ENTER’ method for logistic regression. The final regression model for the 6‒11-month-old group included household size, maternal BMI, maternal food consumption status during last pregnancy, maternal knowledge on consequences of early pregnancy, maternal knowledge on attending at least four antenatal appointments, and whether the child received colostrum as independent variables. The final regression model for the 12‒23-month-old group included household savings status, asset index, maternal BMI, maternal education status, type of antenatal care provider, maternal food consumption status during last pregnancy, maternal knowledge of health care providers, maternal knowledge on exclusive breastfeeding, maternal knowledge on diarrheal management, and the gender of the index child as independent variables. The data analysts were blinded to the identity of the participants (name, addresse and contact number).

## Results

### Descriptive statistics

Data was available for 4,400 children and households in the population targeted by the Suchana program. The descriptive results indicated that 8.1% and 10.3% of children in the 6‒11 and 12‒23-month-old groups suffered moderate wasting, with respective mean weight-for-length z-scores of -2.35 ± 0.28and -2.37 ± 0.26. The difference in proportion of moderate wasting and overall mean WLZ-scores between the age groups were statistically significant (p<0.05). Moreover, 1.57% of 6‒23-month‒olds were suffering SAM, with a mean WLZ-score of -3.73 ± 0.87 (Data not shown).

Among the sampled children, 51% were male and 22.9% and 20.3% of 6‒11 and 12‒23-month-olds were the first child of their parents. In total, significantly more children (p < 0.001) among the 6–11 month- had suffered morbidity within 15 days preceding the survey (56.3% vs 50.4%). In terms of child feeding practices, 85.3% and 83.7% of 6‒11 and 12‒23-month-olds had received colostrum after birth and 87.4% and 86.7% had initiated breastfeeding within one hour after birth. Additionally, 5.8% and 11.9% of children received a minimum acceptable diet (MAD), a critical indicator of feeding practices for young children, in the 6‒11 and 12‒23-month-old groups and the difference in proportion between the groups was statistically significant (p<0.001).

The mean household size was 6.31 ± 2.39 and 6.24 ± 2.37 and median household annual income was Tk. 80,000 (IQR: 55000, 120000) and Tk. 80,000 (IQR: 55025, 120000) for the 6‒11 and 12‒23-month-old groups, respectively. Furthermore, 31.9% and 33.8% of spouses had savings and 13.9% and 14.0% of households with 6‒11 and 12‒23-month-olds were food secure.

The mean maternal age was 26.52 ± 5.72 and 27.46 ± 5.66 years for the 6‒11 and 12‒23-month-old groups and was statistically significant (p<0.001) and mean BMI was around 19.6 kg/m^2^ for both the groups. Among the mothers of the 6‒11 and 12‒23-month-olds, 56.3% and 53.1% washed hands at least during the three critical times.

In terms of maternal reproductive characteristics, 42.8% and 40.4% of mothers in the 6‒11 and 12‒23-month-old groups had received iron and folic acid (IFA) supplementation during their last pregnancy, 15.6% and 14.3% had attended at least four antenatal care (ANC) checkups, 59.8% and 56.3% had visited appropriate institutions for ANC, and 62.8% and 57.8% received ANC from qualified providers. The mean number of maternal pregnancies was 3.23 ± 2.12and 3.27 ± 2.11 for the 6‒11 and 12‒23-month-old groups. Overall, 26.1% and 25.4% of mothers in the 6‒11 and 12‒23-month-old groups reported consuming more than usual or additional food and 33.8% and 35.8% reported resting more than usual during their last pregnancy.

With respect to maternal knowledge, 81.5% and 81.2% of mothers in the 6‒11 and 12‒23-month-old groups knew the importance of IFA supplementation during pregnancy and 82.0% and 83.4% were aware of the importance of children consuming Vitamin A capsules, 43.4% and 45.8% could mention at least one adverse consequence of early pregnancy, 36.8% and 36.0% knew the minimal number of ANC visits required during pregnancy, and 76.3% and 75.9% of mothers identified appropriate health care providers in the community. Additionally, 93.1% and 92.9% knew the importance of initiating of breastfeeding within one hour of birth, 62.8% and 59.6% knew about the importance of exclusive breastfeeding, and 5.3% and 6.1% could describe appropriate diarrheal management for children. The descriptive results of the age groups are presented in Table 2.

**Table 2:** Characteristics of the sample by age groups.

Indicators		Age 6–11, % (*n*) (*N* = 1,200)	Age 12–23, %(n) (*N* = 3,200)	*p*-value
**Child’s characteristics**				
Nutritional status, % (n)				
	Moderate wasting	8.1 (95)	10.3 (324)	0.028
Weight-for-length z-score (moderate wasting), Mean ± SD		-2.35 ± 0.28	-2.37 ± 0.26	0.632
Weight-for-length z-score (Overall), Mean ± SD		-0.54 ± 1.24	-0.88 ± 1.01	<0.001
Gender, % (n)				
	Female	49 (588)	49 (1567)	0.985
	Male	51 (612)	51 (1633)	
Birth order				
	1	22.9 (275)	20.3 (651)	0.162
	2–3	40.2 (482)	43.3 (1385)	
	4–5	24.0 (288)	23.1 (739)	
	6 or above	12.9 (155)	13.3 (425)	
Acute morbidity status in last 15 days, % (n)				
	Had morbidity	56.3 (676)	50.4 (1615)	0.001
**Child feeding practices**				
Received colostrum, % (n)		85.3 (1023)	83.7 (2677)	0.198
Initiation of breastfeeding within 1 hour of birth, % (n)		87.4 (1049)	86.7 (2774)	0.523
Minimum Acceptable Diet, % (n)		5.8 (69)	11.9 (381)	<0.001
Child received any medicine or vitamin drop on the day preceding the survey, % (n)		22.4 (269)	17.5 (560)	<0.001
**Socio-economic status**				
Household size, Mean ± SD		6.31 ± 2.39	6.24 ± 2.37	0.406
Household income (Tk.), Median (IQR)*		80000 (55000, 120000)	80000 (55025, 120000)	0.769
Household savings, % (n)		31.9 (383)	33.8 (1080)	0.250
Asset index				
	1^st^ quintile	21.1 (253)	19.8 (633)	0.168
	2^nd^ quintile	18.8 (226)	21.1 (674)	
	3^rd^ quintile	21.8 (262)	19.3 (618)	
	4^th^ quintile	18.7 (224)	20.0 (640)	
	5^th^ quintile	19.6 (235)	19.8 (635)	
Household Food Insecurity, % (n)				
	Food Secure	13.9 (167)	14.0 (448)	0.931
	Mildly Food Insecure	11.1 (133)	10.8 (347)	
	Moderately Food Insecure	45.7 (548)	46.7 (1493)	
	Severely Food Insecure	29.3 (352)	28.5 (912)	
**General maternal characteristics**				
Maternal age, Mean ± SD (years)		26.52 ± 5.72	27.46 ± 5.66	<0.001
Maternal BMI (Kg/m^2^), Mean ± SD		19.56 ± 3.18	19.58 ± 3.1	0.852
Maternal education status, % (n)				
	No schooling	22.3 (268)	23.9 (765)	0.273
	At least 1 year of formal schooling	77.7 (932)	76.1 (2435)	
Maternal earning status, % (n)				
	Non-earning	97.6 (1171)	96.9 (3102)	0.254
	Earning	2.4 (29)	3.1 (98)	
Handwashing during critical times (at least 3), % (n)		56.3 (676)	53.1 (1699)	0.055
**Maternal reproductive characteristics**				
Had iron-folic acid supplementation during last pregnancy, % (n)		42.8 (513)	40.4 (1293)	0.159
Had at least 4 antenatal care check-ups, % (n)		15.6 (187)	14.3 (457)	0.276
Place of antenatal care visit, % (n)				
	Appropriate	59.8 (718)	56.3 (1802)	0.036
	In-appropriate	40.2 (482)	43.7 (1398)	
Antenatal care provider, % (n)				
	Qualified	62.8 (753)	57.8 (1850)	0.003
	Unqualified	37.3 (447)	42.2 (1350)	
Attendant during last child delivery, % (n)				
	Unqualified	33.3 (400)	31.0 (992)	0.138
	Medically trained	66.7 (800)	69.0 (2208)	
Type of delivery (normal), % (n)		83.9 (1007)	85.1 (2722)	0.346
Place of delivery (home), % *(*n)		76.4 (917)	77.1 (2467)	0.635
Received TT vaccination during pregnancy or before, % (n)		72.5 (870)	73.9 (2366)	0.336
Number of pregnancies, Mean± SD		3.23 ± 2.12	3.27 ± 2.11	0.489
Maternal food consumption status during last pregnancy, % (n)				
	Less than usual	42.3 (507)	44.5 (1425)	0.384
	Same as usual	31.7 (380)	30.1 (963)	
	More than usual	26.1 (313)	25.4 (812)	
Maternal resting status during last pregnancy, % (n)				
	Less than usual	38.2 (458)	36.7 (1174)	0.458
	Same as usual	28.0 (336)	27.5 (880)	
	More than usual	33.8 (406)	35.8 (1146)	
**Maternal knowledge**				
Maternal knowledge of the consumption of IFA supplementation during pregnancy		81.5 (979)	81.2 (2601)	0.819
Maternal knowledge about children consuming Vitamin A capsule		82.0 (984)	83.4 (2671)	0.247
Maternal knowledge of consequence of early pregnancy (at least 1)		43.4 (521)	45.8 (1467)	0.150
Maternal knowledge on attending at least four antenatal care		36.8 (441)	36.0 (1151)	0.631
Maternal knowledge on appropriate health care provider				
	Yes	76.3 (915)	75.9 (2428)	0.795
Maternal knowledge of initiation of breastfeeding with 1 hour after birth		93.1 (1117)	92.9 (2974)	0.866
Maternal knowledge of exclusive breastfeeding		62.8 (753)	59.6 (1906)	0.054
Maternal knowledge of diarrheal management		5.3 (63)	6.1 (195)	0.289

*Kruskal-Wallis equality-of-populations rank test.

### Bi-variate and multivarible analyses

Simple logistic regression models by age showed that children aged 12‒23 months had a 1.3-fold higher risk of moderate wasting compared to 6‒11-month-olds [odds ratio (OR): 1.30 (95% CI: 1.03–1.66); *p* < 0.05; data not shown].

Simple and multiple logistic regression analyses of all children and the children by their age groups are summarized in Table 3. In the 6‒11-month-old group, children from larger households had a 1.11-fold higher risk of moderate wasting [OR: 1.08 (95% CI: 1.01–1.15); *p* < 0.05; adjusted OR (aOR): 1.11 (95% CI: 1.03–1.19), *p* < 0.01]. Higher maternal BMI [OR: 0.85 (95% CI: 0.77–0.94); *p <*0.01; aOR: 0.86 (95% CI: 0.78–0.95), *p <* 0.01] and maternal consumption of more food than usual during the last pregnancy [OR: 0.48 (95% CI: 0.27–0.86); *p* < 0.05; aOR: 0.52 (95% CI: 0.29–0.95), *p* < 0.05] were associated with a lower risk of moderate wasting in the 6‒11-month-old group.

**Table 3:** Risk factors for the occurrence of moderate wasting by age groups.

**6–11-month-olds (n = 1,175)**					
**Variables**		**Unadjusted OR (95% CI)**	**p-value**	*Adjusted OR (95% CI)	**p-value**
Household size		1.08 (1.01, 1.15)	0.029	1.11 (1.03, 1.19)	0.005
Asset index					
	1^st^ quintile	Reference		Reference	
	2^nd^ quintile	0.63 (0.30, 1.31)	0.213	0.69 (0.33, 1.44)	0.323
	3^rd^ quintile	0.51 (0.28, 0.94)	0.031	0.59 (0.32, 1.11)	0.101
	4^th^ quintile	0.52 (0.26, 1.02)	0.058	0.60 (0.30, 1.21)	0.151
	5^th^ quintile	0.50 (0.25, 0.97)	0.041	0.55 (0.27, 1.11)	0.094
Maternal BMI		0.85 (0.77, 0.94)	0.002	0.86 (0.78, 0.95)	0.004
Maternal food consumption status during last pregnancy					
	Less than usual	Reference		Reference	
	Same as usual	0.88 (0.58, 1.33)	0.532	0.83 (0.54, 1.26)	0.378
	More than usual	0.48 (0.27, 0.86)	0.015	0.52 (0.29, 0.95)	0.033
Maternal knowledge on consequence of early pregnancy (at least one)					
	At least 1	Reference		Reference	
	None	1.55 (0.99, 2.40)	0.049	1.40 (0.90, 2.17)	0.133
Maternal knowledge on attending at least four antenatal care					
	No	Reference		Reference	
	Yes	0.56 (0.34, 0.91)	0.021	0.65 (0.40, 1.05)	0.077
The child received colostrum					
	No	Reference		Reference	
	Yes	0.61 (0.38, 0.99)	0.049	0.74 (0.45, 1.24)	0.254
**12–23-month-olds (*n* = 3,153)**					
**Variables**		**Unadjusted OR (95% CI)**	**p-value**	**Adjusted OR (95% CI)**	**p-value**
Household savings					
	No	Reference		Reference	
	Yes	0.78 (0.62, 0.99)	0.039	0.83 (0.65, 1.06)	0.143
Asset index					
	1^st^ quintile	Reference		Reference	
	2^nd^ quintile	0.90 (0.65, 1.25)	0.531	1.00 (0.73, 1.38)	0.985
	3^rd^ quintile	0.59 (0.41, 0.86)	0.007	0.70 (0.47, 1.04)	0.074
	4^th^ quintile	0.75 (0.52, 1.06)	0.102	0.92 (0.62, 1.35)	0.655
	5^th^ quintile	0.63 (0.44, 0.90)	0.011	0.85 (0.56, 1.29)	0.445
Maternal BMI		0.91 (0.87, 0.96)	<0.001	0.93 (0.89, 0.98)	0.004
Maternal education status					
	No schooling	Reference		Reference	
	At least 1 year of formal schooling	0.74 (0.57, 0.95)	0.020	0.86 (0.66, 1.11)	0.247
Antenatal care provider					
	Qualified	Reference		Reference	
	Unqualified	1.33 (1.05, 1.68)	0.019	1.13 (0.87, 1.48)	0.360
Maternal food consumption status during last pregnancy					
	Less than usual	Reference		Reference	
	Same as usual	0.77 (0.59, 1.00)	0.051	0.75 (0.58, 0.99)	0.039
	More than usual	0.75 (0.58, 0.98)	0.037	0.82 (0.63, 1.08)	0.152
Maternal knowledge on health care provider					
	Appropriate	Reference		Reference	
	In-appropriate	1.33 (1.01, 1.75)	0.045	1.23 (0.93, 1.64)	0.141
Maternal knowledge on exclusive breastfeeding					
	No	Reference		Reference	
	Yes	0.78 (0.62, 0.98)	0.031	0.84 (0.66, 1.07)	0.150
Maternal knowledge on diarrheal management					
	No	Reference		Reference	
	Yes	0.46 (0.25, 0.86)	0.015	0.50 (0.27, 0.93)	0.028
Gender of the index child					
	Male	Reference		Reference	
	Female	0.71 (0.56, 0.89)	0.004	0.72 (0.57, 0.90)	0.004
**6–23-month-olds (*n* = 4,331)**					
Asset index					
	1^st^ quintile	Reference		Reference	
	2^nd^ quintile	0.83 (0.61, 1.13)	0.251	0.90 (0.68, 1.20)	0.485
	3^rd^ quintile	0.57 (0.40, 0.80)	0.001	0.65 (0.47, 0.93)	0.018
	4^th^ quintile	0.69 (0.50, 0.94)	0.019	0.84 (0.62, 1.16)	0.291
	5^th^ quintile	0.60 (0.43, 0.83)	0.002	0.79 (0.56, 1.11)	0.173
Maternal age		1.02 (1.01, 1.04)	0.014	1.02 (1.00, 1.04)	0.066
Maternal BMI		0.91 (0.86, 0.94)	<0.001	0.91 (0.87, 0.95)	<0.001
Maternal education status					
	No schooling	Reference		Reference	
	At least 1 year of formal schooling	0.71 (0.57, 0.89)	0.004	0.90 (0.71, 1.16)	0.434
Antenatal care provider					
	Qualified	Reference		Reference	
	Unqualified	1.30 (1.04, 1.61)	0.022	1.11 (0.88, 1.42)	0.368
Maternal food consumption status during last pregnancy					
	Less than usual	Reference		Reference	
	Same as usual	0.80 (0.64, 0.97)	0.027	0.80 (0.64, 0.98)	0.031
	More than usual	0.68 (0.54, 0.87)	0.003	0.75 (0.59, 0.95)	0.020
Maternal knowledge on health care provider					
	Appropriate	Reference		Reference	
	In-appropriate	1.30 (1.03, 1.65)	0.028	1.21 (0.95, 1.53)	0.113
Maternal knowledge on exclusive breastfeeding					
	No	Reference		Reference	
	Yes	0.76 (0.61, 0.94)	0.011	0.81 (0.65, 1.00)	0.058
Maternal knowledge on diarrheal management					
	No	Reference		Reference	
	Yes	0.48 (0.28, 0.83)	0.009	0.51 (0.30, 0.87)	0.014
Gender of the index child					
	Male	Reference		Reference	
	Female	0.72 (0.58, 0.89)	0.003	0.72 (0.58, 0.89)	0.003
Child received any medicine or vitamin drop on the day preceding the survey					
	No	Reference		Reference	
	Yes	1.56 (1.22, 1.99)	0.001	1.67 (1.30, 2.13)	<0.001

* Please refer to Table 1 for definitions of abbreviations.

**Adjusted for all variables included in the multivariable model.

Higher maternal BMI [OR: 0.91 (95% CI: 0.87–0.96); *p* < 0.001; aOR: 0.93 (95% CI: 0.89–0.98), *p* < 0.01], consumption of a normal amount of food during the last pregnancy [OR: 0.77 (95% CI: 0.59–1.00); *p* = 0.051; aOR: 0.75 (95% CI: 0.58–0.99), *p* < 0.05], and having maternal knowledge of diarrheal management [OR: 0.46 (95% CI: 0.25–0.86); *p* < 0.05; aOR: 0.50 (95% CI: 0.27–0.93), *p* < 0.05] were associated with a significantly lower risk of moderate wasting among 12‒23-month-olds. Being female [OR: 0.71 (95% CI: 0.56–0.89); *p* < 0.01; aOR: 0.72 (95% CI: 0.57–0.90), p < 0.01] was associated with a significantly lower risk of moderate wasting among 12‒23-month-olds. The results are shown in Table 3 by their age groups and for all 6‒23-month-olds.

**Fig 1:**
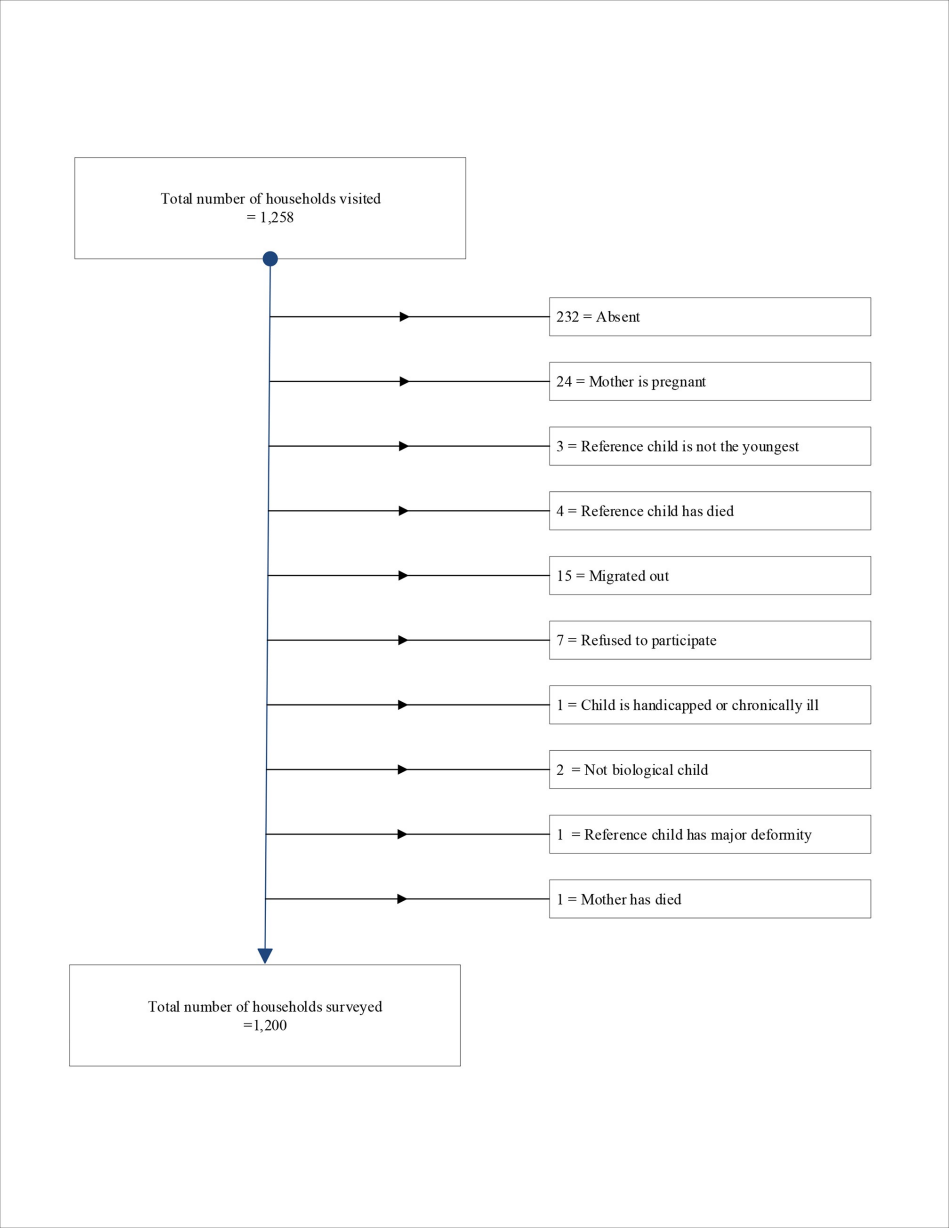
Trial profile for 6-11-month-old children.

**Fig 2:**
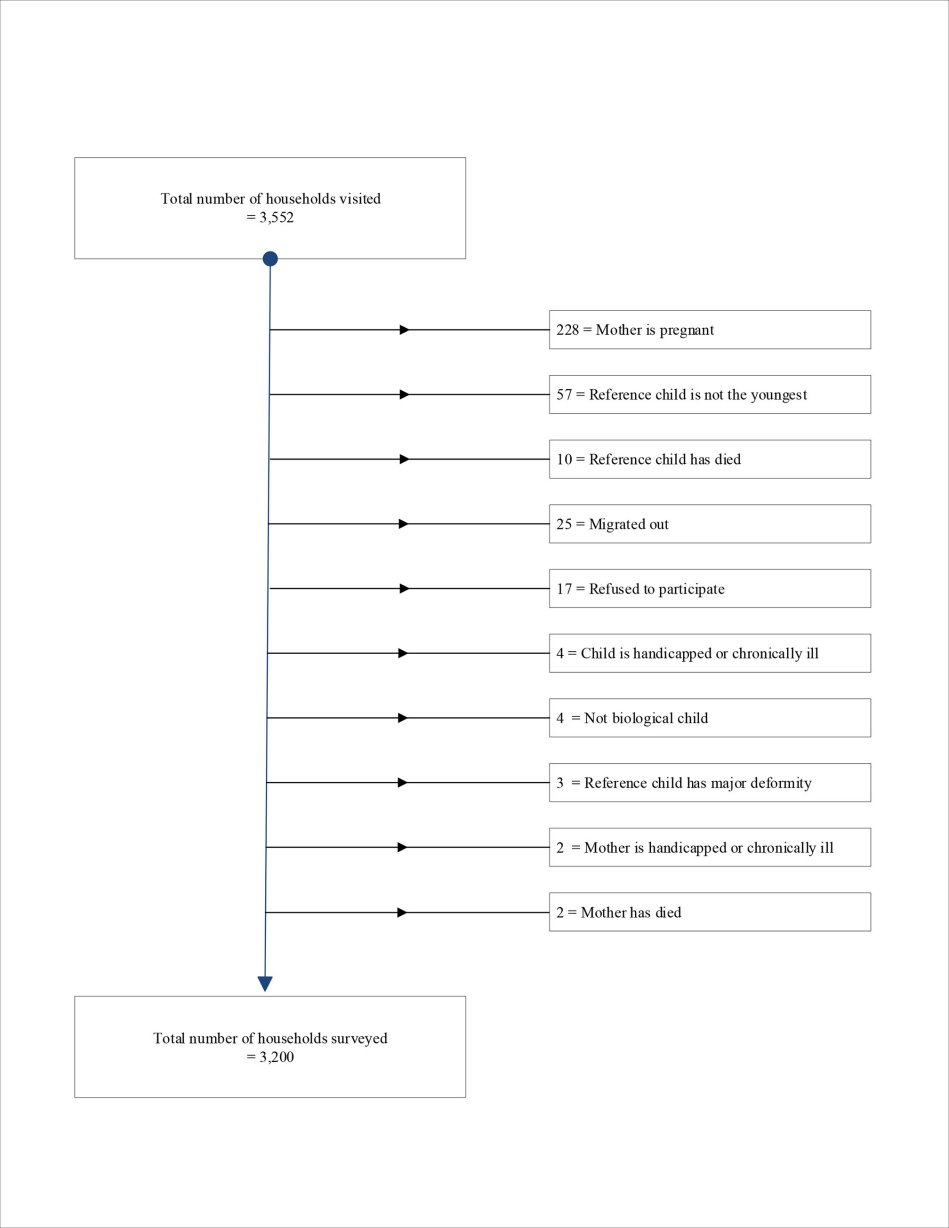
Trial profile for 12-23-month-old children.

In respect of the post-diagnostic tests, all variables used in the multiple regression models for the 6‒11-month and 12‒23-month-old groups had VIF of around 1.0 (mean VIF: 1.05 and 1.06), suggestive of minimum multicollinearity. The overall predictive accuracy was 91.9% for the 6‒11-month-old group regression model and 89.7% for the 12‒23-month-old group, indicating good predictive accuracy of models as well as absence of significant multicollinearity. When considering all children, the asset index, maternal BMI, maternal food consumption status during last pregnancy, maternal knowledge of diarrheal management, the gender of the index child, and consumption of any medicine or vitamin drops by child on the previous day were statistically significant factors associated with moderate wasting. The trial profiles for the two age groups are presented in Figs 1 and 2.

## Discussion

The purpose of this study was to identify the factors associated with the occurrence of moderate wasting among children under two-years-old in the Suchana program catchment households in rural Bangladesh.

Positive associations between increased maternal food consumption during pregnancy and adequate pregnancy weight gain with birth outcomes and subsequent growth and development and association between maternal nutrition during pregnancy and fetal growth have been documented in the literature [29, 30]. Fetal growth retardation due to sub-optimal nutrient intake during pregnancy may lead to a low birthweight, which is a critical risk factor for growth delay during the first two years of life [29, 31]. Hence, increased maternal food consumption during pregnancy may protect younger children from moderate wasting.

Additionally, we have found that higher maternal BMI was significantly associated with moderate wasting in both the age groups, as previously observed in several studies in Bangladesh [19, 26, 32–34]. Appropriate maternal BMI and weight gain during pregnancy are associated with positive fetal outcome [35], indicating optimal maternal nutrition status is associated with positive birth outcomes [35, 36]. Assuming that current maternal BMI reflects maternal BMI status during pre-pregnancy, our findings indicate that the protective effect of a higher BMI on moderate wasting among children is sustained over a long period, and hence higher maternal BMI could protect children from becoming malnourished. Furthermore, mothers with a low BMI or who are undernourished may not be able to produce and provide sufficient amounts of breastmilk for their infants, which may be a critical factor that leads to acute malnourishment among children [20].

Over 8% of 6‒11-month-olds and 10% of 12‒23-month-olds were suffering from moderate wasting among the studied marginalized population, which is similar to the contemporary prevalence of moderate wasting among Bangladeshi under-fives of around 14% among the general population [13, 19]. However, it should be noted that the Bangladesh Demographic and Health Survey was published a few years before the collection of the Suchana data, and the prevalence of wasting in Bangladesh is 8% currently [37]. Moreover, the proportion of children receiving a MAD and the proportion of food secure households were both lower than the national figures [13]; thus, there is scope to alleviate malnutrition in the region via improving MAD and food security status.

Children aged 12‒23 months were significantly more likely to suffer moderate wasting than 6‒11-month-olds, in confirmation of similar findings that older age was significantly associated with moderate wasting in children [3, 19]. However, our findings did not demonstrate a significant association between various child, maternal and socio-economic factors that were previously associated with wasting in other studies [32, 38]; this variation could be due to the differences between the study population, sampling methods or settings and underlying mechanisms. The population of this study was the most vulnerable rural households that are severely affected by difficulties related to poverty and health in Bangladesh. Thus, while poverty or low socio-economic status are often a risk factor for undernutrition [32], it did not come up as a significant factor in our study which could be due to selection of the most vulnerable households in the communities. Therefore, associated factors in general population might thus be different.

Previous literature found that children’s nutrition status is associated with food availability in the household [32]. Larger families often have less food available per head compared to smaller families [39, 40]. In addition, vulnerable members of larger households have a higher risk of acquiring infectious diseases due to overcrowding and the comparatively limited access of larger families to healthcare services, which may further negatively impact the nutrition status of young members of larger households [39–42]. Thus, large nutrition programs like Suchana should address disparity in intra-household food distribution, promote optimal food intake and aid in food availability for vulnerable members of larger households.

Our analyses involving wasting were based on the WHO suggested cut-off values using weight-for-length z-score. The literature suggests that while both diagnose wasting, MUAC and WLZ-score are both independent indicators: MUAC is primarily used as a screening tool at the community level and WLZ-score is used in health care facilities. There is a poor correlation between MUAC and WLZ-score, calculations have shown that cut-off values for detecting moderate wasting using MUAC perform poorly compared to the same populations defined by WLZ-score [43].

An analysis of the cost-effectiveness of interventions for maternal and child health outcomes in terms of cost per death averted showed that maintaining optimal nutrition during pregnancy was the most expensive intervention [44]. Nonetheless, Suchana should focus on educating mothers on the importance and benefits of maintaining optimal nutrition during pregnancy for their own and their child’s health outcomes.

Multiple studies [39, 45, 46] suggest maternal knowledge of nutrition and health is significantly associated with children’s nutrition status and this knowledge can be acquired through different channels other than educational institutions [47]. Maternal knowledge of diarrheal management could be viewed as a proxy indicator for knowledge on the management of common ailments. The significant association between maternal knowledge of diarrheal management and moderate wasting in this study suggests mothers with knowledge of diarrheal management might have been able to prevent or reduce the duration of diarrheal and other common illnesses among their children, which may in turn reduce the incidence of infections, an important risk factor for moderate wasting. Knowledge of diarrheal management have may have been associated with reduced diarrhea in children though an indirect pathway by being linked to other positing health care behaviors which may have been associated with reduced diarrhea and wasting. While the proportion of mothers with knowledge of diarrheal management was only around 6%, this factor appeared to be potentially protective against wasting, indicating that raising awareness among mothers on diarrheal management could be a potentially useful major intervention to prevent wasting. Suchana has the scope to disseminate information on the causes and management of acute malnutrition among the members of the beneficiary households and we hope the mothers in the catchment areas will acquire additional knowledge that may help to combat child malnutrition.

Gender emerged as a significant factor associated fwith moderate wasting: male children in the older age group were significantly more vulnerable than females. Though similar findings on wasting and undernutrition have been repeatedly documented by similar studies [38, 39, 42, 48], the mechanisms that underlie this relationship are unclear and warrant further investigation as a robust and acceptable scientific explanation is yet to be established. However, it is often argued that the vulnerability of young male children reflects a natural process [49].

The infection-malnutrition cycle [50] suggests malnutrition and morbidity in children are closely related; over half of all children were reported to have suffered some illness in the 15 days prior to the survey. However, we did not observe a relationship between moderate wasting and morbidity in this study; the absence of an association may be due to the point prevalence survey with short recall period.

National guidelines have been published for the community management of moderate wasting in Bangladesh [14] and activities could be planned in the community to manage moderate wasting based on these guidelines. The factors identified to be significantly associated with moderate wasting in our analyses will help to revise program targets, enable course corrections, and assist with formulation of new strategies or modification of the existing intervention components of the Suchana or similar programs, if necessary. The marginalized population examined in this study was mostly food insecure and thus requires tailored interventions with short and long term planning through Suchana, which may be different to the interventions required to manage and prevent moderate wasting in food secure populations [15].

### Limitations

The relationship between seasonality in Bangladesh and the occurrence of moderate wasting was not examined in this study; nonetheless, the occurrence of moderate wasting does not have a peak season in Bangladesh. We have only analyzed cases confirmed to have acute malnutrition as defined by the WLZ-score cut-off value, inclusion of cases defined as acutely malnourished by MUAC cut-off value, may have given us the opportunity to analyze the full spectrum of cases that can be considered as moderate acute malnutrition. The recall period for acute morbidity was only 15 days, thus this variable does not indicate the duration of the morbidity episodes. Causality cannot be inferred due to the cross-sectional nature of this study; hence, only association between variables were reported. The Suchana program included marginalized households only, thus the inferences derived from this study was applicable to the marginalized sup-group and may not necessarily represent determinant factors among the general population of Bangladesh. Finally, information collected through maternal responses may be prone to recall bias. Nonetheless, the systematic sampling and robust sample size ensured that potential recall bias is diluted.

### Ethical approval

This study was approved by the Research Review Committee and Ethical Review Committee, the two obligatory components of the institutional review board of International Centre for Diarrhoeal Disease Research, Bangladesh (icddr,b) under research protocol number PR-16020. Informed written consent was obtained from the parents of all children. The respondents were also informed that their participation was voluntary and of their right to withdraw themselves from the study at any point for any reason. Information that could reveal any respondent’s identity was not used while analyzing the data. The Suchana evaluation study has been registered at the Registry for International Development Impact Evaluations (RIDIE) as RIDIE-STUDY-ID-5d5678361809b.

## Supporting information

S1 File Questionnaires. (DOCX)

S1 Data (XLS)
