# Supplementary material for: Factors associated with moderate wasting among marginalized 6 to 23-month aged children in Bangladesh: Findings of the Suchana program baseline survey data
Source: PLoS One. 2020 Aug 20;15(8):e0236786. doi: 10.1371/journal.pone.0236786 (PMC7440651; doi:10.1371/journal.pone.0236786)
Supplement: S1 File — (DOCX) [file pone.0236786.s001.docx]

**Evaluation of the impact of *Suchana* on preventing chronic malnutrition: a cluster randomized trial**

**Household Questionnaire
Nutrition and Clinical Services Division, icddr,b**

Child age group: 0-5mo=1, 6-11mo=2, 12-23mo=3

Enumerator’s name: Code: ..

Cross-checked by: .. Code: ..

Supervised by: .. Code: ..

If interview cannot be taken, what is the reason?

Main female not available[1];Refused to participate [2];others, specify.... ... ... .. .

**H1. Household identification**

| **No.** | **Household identification** | **Response** | | | | | | | | | | | | **No.** | **Household identification** | **Response** | | | |
| --- | --- | --- | --- | --- | --- | --- | --- | --- | --- | --- | --- | --- | --- | --- | --- | --- | --- | --- | --- |
| **1.1** | Household Identification Number: |  | |  |  |  |  | |  | | | | | **1.6** | Household’s ethnic group:  Bangalee 1  Tribal (specify) 2  Other (specify 3 |  |  |  | |
|  |  |  | | | | | | |  |  |  |  |  |  |  |  |  |  |  |
|  |  |  |  |  |  |  |  |  |  |  |  |  |  |  |  |  |  |  |  |
| **1.2** | Beneficiary/member ID (Listed by Suchana) |  | | | | | | | | | | | | **1.7** | Date of the first visit (dd/mm/yy): |  | | |  |
|  |  |  |  |  |  |  |  |  |  |  |  |  |  |  |  | day | month | year |  |
|  |  |  |  |  |  |  |  |  |  |  |  |  |  |  |  |  |  |  |  |
|  |  |  |  |  |  |  |  |  |  |  |  |  |  |  |  |  |  |  |  |
|  |  |  |  |  |  |  |  |  |  |  |  |  |  |  |  |  | | |  |
|  |  |  | | | | | | | | | | | |  |  |  |  |  |  |
| **1.3** | Beneficiary contact number: |  |  | | | | | | | | | | | **1.8** | Date of the second visit (dd/mm/yy): |  | | |  |
|  |  |  |  |  |  |  |  |  |  |  |  |  |  |  |  | day | month | year |  |
|  |  |  |  | | | | | | | | | | |  |  |  |  |  |  |
|  |  |  |  |  |  |  |  |  |  |  |  |  |  |  |  |  | |  |  |
|  |  |  |  | | | | | | |  | | | |  |  |  |  |  |  |
| **1.4** | GPS coordinates: |  |  |  |  |  |  |  |  |  | | | | **1.10** | Name of data verifier and code: |  |  |  | |
|  |  |  | |  | |  |  |  | |  |  |  |  |  |  |  | |  |  |

**H2. Household characteristics**

| Line # | Name | Sex  Male  =1  Female  =2 | Father line  number  (of the  child)  Dead= 98  Lives  Elsewhere= 99 | Relation  to  household  head | Age | | Marital  Status | Age at  marriage | Religion | Language  Bengali=1  Indigenous=2 | Literacy | Education  (Highest  class  passed) | Type of School | NGO member Yes=1 No=0 | Occupation | | Monthly income from main source (last month) | Monthly income from all sources (last month) | Yearly  income  from  all  sources |
| --- | --- | --- | --- | --- | --- | --- | --- | --- | --- | --- | --- | --- | --- | --- | --- | --- | --- | --- | --- |
|  |  |  |  |  |  |  |  |  |  |  |  |  |  |  | Main | Secondar  y |  |  |  |
| 2.1 | 2.2 | 2.3 | 2.4 | 2.5 | 2.6 | | 2.7 | 2.8 | 2.9 | 2.10 | 2.11 | 2.12 | 2.13 | 2.14 | 2.15 | 2.16 | 2.17 | 2.18 | 2.19 |
|  |  |  |  |  | Skip to next if  age is less  below 5 years | |  |  |  |  |  | Skip to  2.14 if  (88) |  |  |  |  |  |  |  |
|  |  | Code ↑ | Code ↑ | Code 1 | Year | Month | Code 2 | Years | Code 3 | Code ↑ | Code 4 | Code 5 | Code  5a | Code ↑ | Code  6 | Code 6 | Tk. | Tk. | Tk. |
|  |  |  |  |  |  |  |  |  |  |  |  |  |  |  |  |  |  |  |  |
|  |  |  |  |  |  |  |  |  |  |  |  |  |  |  |  |  |  |  |  |
|  |  |  |  |  |  |  |  |  |  |  |  |  |  |  |  |  |  |  |  |
|  |  |  |  |  |  |  |  |  |  |  |  |  |  |  |  |  |  |  |  |
|  |  |  |  |  |  |  |  |  |  |  |  |  |  |  |  |  |  |  |  |
|  |  |  |  |  |  |  |  |  |  |  |  |  |  |  |  |  |  |  |  |
|  |  |  |  |  |  |  |  |  |  |  |  |  |  |  |  |  |  |  |  |
|  |  |  |  |  |  |  |  |  |  |  |  |  |  |  |  |  |  |  |  |
|  |  |  |  |  |  |  |  |  |  |  |  |  |  |  |  |  |  |  |  |
|  |  |  |  |  |  |  |  |  |  |  |  |  |  |  |  |  |  |  |  |
|  |  |  |  |  |  |  |  |  |  |  |  |  |  |  |  |  |  |  |  |

**Code list for Section B:**

| **Code 1: Relationship** |  | **Code 5: Education (Highest class passed)** |  | **Code 6: Main/Secondary Occupation** |
| --- | --- | --- | --- | --- |
| **Relationship with HH head** |  | Never attended school 86 |  | **Day Labor** |
| Mother of HH head | 1 | Reads in class I 0 |  | Agricultural (rice/paddy) [1] |
| Spouse of HH head | 2 | Completed class I 1 |  | Agricultural (other than rice/paddy) [2] |
| Son/daughter | 3 | Completed class II 2 |  | Unskilled labor [3] |
| Daughter/son -in-law | 4 |  |  | Skilled labor [4] |
| Grandson/daughter | 5 | Put number of highest completed class. |  | House maid [5] |
| Father of HH head | 6 | For example, if currently in class IV, put 3 |  | Works in restaurant/tea stall [6] |
| Brother/sister | 7 | (class III completed) |  | Other (repair/construction) [7] |
| Niece/Nephew | 8 |  |  | Other (specify) [8] |
| Household head’s cousin | 9 | Completed Secondary School 10 |  | **Self-employment** |
| Household head | 10 | Completed Higher Secondary 12 |  | Rickshaw/van pulling [9] |
|  |  | Graduate or above 44 |  | CMG/Tempu/Mini bus driver [10] |
| **In-laws of primary respondent** |  | Preschool class (general) 55 |  | Bus/Truck driver [11] |
| Father-in-law/mother-in-law | 11 | Preschool (mosque based) 66 |  | Beggar (professional) [12] |
| Brother/Sister-in-law | 12 | Other (specify) 77 |  | Transport workers [13] |
| Spouse's niece/nephew | 13 |  |  | Teacher [14] |
| Spouse’s cousin | 14 |  |  | **Production** |
|  |  |  |  | Food Processing [15] |
|  |  | **Code 5a: Type of School** |  | Small industry [16] |
| **Other relative/non relative** |  | Public/ govt. school/ college | 1 | Handicrafts [17] |
| Other relative | 15 | Semi govt. school/college | 2 | **Trading** |
| Permanent servant | 16 | Private school/ college | 3 | Small trader (roadside stand or stall) [18] |
| Other Non relative/friends | 17 | Madras- Board curriculum | 4 | Small trader (village or village market) [19] |
|  |  | Madras- Koami/ only religious curriculum | 5 | Other trading [20] |
|  |  | Don’t know | 88 | **Farming** |
| **Code 2: Marital status code** |  |  |  | Field crop farming [21] |
| Unmarried (never married) 1 |  |  |  | Homestead farming [22] |
| Married 2 |  |  |  | Raising fish / fish pond [23] |
| Widow/widower 3 |  |  |  | Fishrman [24] |
| Divorced 4 |  |  |  | Raising poultry [25] |
| Separated/Deserted 5 |  |  |  | Raising livestock [26] |
|  |  |  |  | **Non-earning occupation** |
|  |  |  |  | Housewife [27] |
| **Code 3: Religion** |  |  |  | Do nothing [28] |
| Muslim 1 |  |  |  | Student [29] |
| Hindu 2 |  |  |  | Children less than 5 years of age [30] |
| Christian 3 |  |  |  |  |
| Buddhist 4 |  |  |  |  |
| Other (specify) 5 |  |  |  |  |
| **Code 4 : Literacy** |  |  |  |  |
| Cannot read and write. 1 |  |  |  |  |
| Can sign only 2 |  |  |  |  |
| Can read only 3 |  |  |  |  |
| Can read and write 4 |  |  |  |  |

**H3. Household status**

| 3.1.1 | Ownership of the house. | 1=Own  2=Rented  3=Living in kind/*khas* 4=Living illegally |  |  |  |
| --- | --- | --- | --- | --- | --- |
|  |  |  |  |  |  |
|  |  |  |  |  |  |
| 3.1.2 | How many rooms are there in this household? | Exact no. of rooms |  |  |  |
|  |  |  |  |  |  |
| 3.1.3 | How many rooms are used for sleeping? | Exact no. of rooms |  |  |  |
|  |  |  |  |  |  |
| **Materials used for house construction (Observation Only)** | | | | | |
| 3.2.1 | Floor Materials | 1=Natural Floor (clay/earth/sand)  2=Rudimentary floor (Wood planks /Palm/bamboo) 3=Finished Floor (Wooden /Cement)  4=Others |  |  |  |
|  |  |  |  |  |  |
|  |  |  |  |  |  |
| 3.2.2 | Roof Materials | 1=Natural roofing (straw/leaves)  2=Rudimentary roofing (Wood planks, Palm/bamboo Cardboard Thatch roof Chatai’ roof)  3=Finished roofing [Tin ,Wood, Tali (traditional tiles)] 4=Others |  |  |  |
|  |  |  |  |  |  |
|  |  |  |  |  |  |
|  |  |  |  |  |  |
| 3.2.3 | Exterior Walls Materials | 1=Natural wall ( No walls Cane/palm/trunks,Dirt) 2=Rudimentary walls (Bamboo with mud ,Stone with mud, Plywood, Cardboard)  3=Finished walls (Tin, Cement , Stone with lime/cement Bricks , Wood planks/shingles)  4=Others |  |  |  |
|  |  |  |  |  |  |
|  |  |  |  |  |  |
| 3.2.4 | What is the present main source of lighting for this house? | 1=Kerosene lamp/lantern/petromax 2= Electricity  3=Candle  4=Gas lamp/bio gas  5=Solar energy/light  6=Other (specify) |  |  |  |
|  |  |  |  |  |  |
|  |  |  |  |  |  |

| 3.2.5 | What is the current main source of cooking fuel of this household? | 1= Electricity  2= LPG  3= Natural gas  4= Biogas  5= Kerosene  6= Charcoal  7= Wood  8= Straw/shrubs/grass  9= Agricultural crop residual 10= Animal dung  11= Others |  |
| --- | --- | --- | --- |
| **Remittance Consultant’s Line number#** | | | |
| 3.3.1 | Did anyone send remittances back to the household during last 1 year? | 1=Yes  0=No  88=Don’t know  (skip to 3.4.1 if ‘0’ or ‘88’) |  |
| 3.3.2 | Is this money coming from within Bangladesh or from another country? And how much was received in the last 1 year (if the respondent doesn’t know the amount code 888888) | Bangladesh Another country |  |
| **Schooling** | | | |
| 3.4.1 | Any children in the household aged between 5-7 years enrolled in school/madrasa within last 1 year | 1=Yes  0=No  (skip to H4.1 if ‘0’) |  |
| 3.4.2 | If ‘Yes’ in Q.11. How many |  |  |

**H4. Presence of capital items H4.1. Business assets**

**Consultant’s Line number#**

| Does your HH own any of these business assets (including those given away for rearing)? | | | | | | | | | Do you rear any animals/fish that you don’t own? | | | During the last year, which of the following business assets did you sell or give away? | | | |
| --- | --- | --- | --- | --- | --- | --- | --- | --- | --- | --- | --- | --- | --- | --- | --- |
| Sl. | a. Asset | b.  Number (skip to ‘i’ if ‘0’) | c. For how long do you have this asset  (mention the item you have for the longest period)  Months.  Years | d. How much is this  asset worth today?  (Taka) | e. How did you acquire this  asset?  (code 1) (skip to ‘g’ if any code  except  opt. 1) | f. If purchased, how did you finance the purchase? (code 2) | g. Is this asset on sharing??  Yes [1]  No [0]  (skip to ‘i’ if ‘0’) | h. If shared, please mention the percentage shared  Percentage  (%) | i.  Number (skip to ‘l’ if ‘0’) | j. For how  long do you have this asset (mention the item you have for the longest period)  Months | k. From whom/where did you acquire it?  (code 3) | l.  Number (skip to next if ‘0’) | m. How much is this asset  worth when you sold it?  (Taka) | n. How much is this  asset worth today?  (Taka) | o. How did you transfer this  asset?  Sold it [1];  Dowry [2];  Gifted [3];  Others (specify) [4]  *Multiple  answer |
| 4.1.1 | Cows |  |  |  |  |  |  |  |  |  |  |  |  |  |  |
| 4.1.2 | Chickens and Ducks |  |  |  |  |  |  |  |  |  |  |  |  |  |  |
| 4.1.3 | Birds e.g. pigeons |  |  |  |  |  |  |  |  |  |  |  |  |  |  |
| 4.1.4 | Goats/ sheep |  |  |  |  |  |  |  |  |  |  |  |  |  |  |
| 4.1.5 | Pigs |  |  |  |  |  |  |  |  |  |  |  |  |  |  |
| 4.1.6 | Fish |  |  |  |  |  |  |  |  |  |  |  |  |  |  |
| 4.1.7 | Others  animals/birds (specify) |  |  |  |  |  |  |  |  |  |  |  |  |  |  |
| 4.1.8 | Plough |  |  |  |  |  |  |  |  |  |  |  |  |  |  |
| 4.1.9 | Mowing machine |  |  |  |  |  |  |  |  |  |  |  |  |  |  |
| 4.1.10 | Unit for keeping livestock (cattle house) |  |  |  |  |  |  |  |  |  |  |  |  |  |  |
| 4.1.11 | Shop premises |  |  |  |  |  |  |  |  |  |  |  |  |  |  |
| 4.1.12 | Unit for storing crops |  |  |  |  |  |  |  |  |  |  |  |  |  |  |
| 4.1.13 | Boat |  |  |  |  |  |  |  |  |  |  |  |  |  |  |
| 4.1.14 | Boat with a motor |  |  |  |  |  |  |  |  |  |  |  |  |  |  |
| 4.1.15 | Fishnet |  |  |  |  |  |  |  |  |  |  |  |  |  |  |
| 4.1.16 | Rickshaw /van |  |  |  |  |  |  |  |  |  |  |  |  |  |  |
| 4.1.17 | Trees (above 100 Tk) |  |  |  |  |  |  |  |  |  |  |  |  |  |  |
| 4.1.18 | Cart |  |  |  |  |  |  |  |  |  |  |  |  |  |  |
| 4.1.19 | Sewing machine |  |  |  |  |  |  |  |  |  |  |  |  |  |  |
| 4.1.20 | CNG/*Nosimon* |  |  |  |  |  |  |  |  |  |  |  |  |  |  |

| 4.1.21 | Others |  |  |  |  |  |  |  |  |  |  |  |  |  |  |
| --- | --- | --- | --- | --- | --- | --- | --- | --- | --- | --- | --- | --- | --- | --- | --- |
| 4.1.22 | Others |  |  |  |  |  |  |  |  |  |  |  |  |  |  |
| **code 1:** Purchased [1]; Inherited [2]; Gifted [3]; Dowry [4]; Built [5]; Suchana which includes local IPs and coalition members [6]; Other NGO [7], Government [8]; Acquisition following production-sharing [9]; Other (specify)  [10]____________________________ | | | | | | | | | | | | | | | |
| **code 2:** Own savings [1]; Cash from Dowry which includes local IPs coalition members | | | [2]; Borrowed from relatives [3]; Borrowed from [9]; Other [10] | | | friends/neighbours [4]; Borrowed from local NGO [5]; Borrowed from other NGO [6];Borrowed from bank [7]; Borrowed from money lender [8]; Suchana | | | | | | | | | |
|  |  |  |  | | |  |  |  |  |  |  |  |  |  |  |
| **code 3:** Other NGO [1]; Brother/Sister [2]; Relatives [3]; Friends/relatives [4]; Suchana [5]; Others (specify) [6] | | | | | | | | | | | | | | | |

**H4.2. Non-business assets**

How many, if any, do you own of each type of these assets, fill in number.

|  | Assets | Number of assets  (skip to next if ‘0’) | Value of assets  (Taka) | How did you acquire this?  *Multiple answer  Purchased [1]; Inherited [2]; Gifted  [3]; Other [4] |
| --- | --- | --- | --- | --- |
| 4.2.1. | Radio/cassette player/DVD/CD player |  |  |  |
| 4.2.2. | Television |  |  |  |
| 4.2.3. | Electric fan |  |  |  |
| 4.2.4. | Mobile phone |  |  |  |
| 4.2.5. | Bicycle |  |  |  |
| 4.2.6. | Motorcycle |  |  |  |
| 4.2.7. | Chair |  |  |  |
| 4.2.8. | Table |  |  |  |
| 4.2.9. | Chouki (bed thing) |  |  |  |
| 4.2.10. | Sofa (any type) |  |  |  |
| 4.2.11. | Mosquito net |  |  |  |
| 4.2.12. | Jewellery |  |  |  |
| 4.2.13. | Ceremonial sarees for main woman |  |  |  |
| 4.2.14. | Other |  |  |  |
| 4.2.15. | Other |  |  |  |

**H5. Land and water bodies**

**Consultant’s Line number#**

| Note: List all land (all types of land & water bodies) owned or under operation by the household in last 12 months | | | | | | | | | | |
| --- | --- | --- | --- | --- | --- | --- | --- | --- | --- | --- |
| Sl. # | Description code  Homestead 1  Cultivable/arable land 2  Pasture 3  Bush/forest 4  Cultivable Pond 5  Derelict pond ^6^  Waste/non-arable land 7  Land in riverbed 8  Other residential/commercial plot 9 | a.What is  produced with  the asset?  Code 1 | b.Owner =  HH member line number  Owned jointly =66  Outside HH private land=77  Khas/gov land/other  institutions=88  (if code 88; skip ‘g’ only) | c.Size/  Area  (Decimal) | d.Current  operational  status?  (Code 2) | e.Operational status in last season (as per crop calender)?  (Code 2)  (code 2)  (skip to ‘g’ if not  ‘3’ or ‘6’) | f.If the plot is rented/ leased out for  cash, report amount received in the  last season for production.  (Taka) | g.Current market value of the land (amount expect to receive if you sold today)  (Taka) | h.Duration of  owning this  property? | |
|  |  |  |  |  |  |  |  |  | Year | Month |
| 1_._ | Homestead |  |  |  |  |  |  |  |  |  |
|  |  |  |  |  |  |  |  |  |  |  |
|  |  |  |  |  |  |  |  |  |  |  |
|  |  |  |  |  |  |  |  |  |  |  |
|  |  |  |  |  |  |  |  |  |  |  |
|  |  |  |  |  |  |  |  |  |  |  |
|  |  |  |  |  |  |  |  |  |  |  |
|  |  |  |  |  |  |  |  |  |  |  |
|  |  |  |  |  |  |  |  |  |  |  |
| Code 1: Cereals [1]; Vegetable [2]; Fruits [3]; Fish [4]; Chicken/duck [5]; Livestock [6]; Others (specify) _[7];_ Nothing [8] | | | | | | | | | | |
| Code 2: Fallow [1]; Own operated [2]; Rented/leased in (cash) [3]; Rented/leased in (crop share) [4]; Mortgaged in [5]; Rented/leased out (cash) [6]; Rented/leased out (crop share) [7]; Mortgage out [8] | | | | | | | | | | |

**H6. Toilets, water sources, water management, food and environmental hygiene**

| 6.1 | Do you have a latrine? | Yes [1]; No [2]; Yes but shared [3] | |  |
| --- | --- | --- | --- | --- |
| 6.2 | What type of latrine is it? | 1=Piped sewer system; 2=Septic tank; 3=Pit toilet with slab; 4=Pit toilet without slab; 5=Ring slab with water seal; 6=Ring slab without water seal; 6= Hanging toilet; 8=Here and there | |  |
|  |  |  |  |  |
|  |  |  |  |  |
| 6.3 | Where do you dispose the following waste?  **Codes:** Specific place (inside or outside backyard) [1]; Here and there [2]; In a specific Toilet [4]; Others (specify) [5] | | disposal facility [3];  Kitchen Children feces  Poultry waste Livestock waste Domestic waste | Where  \|___\| \|___\| \|___\| \|___\| \|___\| |
|  |  | |  |  |
| **Hand washing location (spot check)** | | | | |
| 6.4 | Specific Place for handwashing (where water and soap/ash/mud is available) | | Yes=1, No= 0 |  |
| 6.5 | Within 30 feet from the toilet structure | | Yes=1, No= 0 (skip to ‘6.8’ if ‘0’) |  |
| 6.6 | With water available within 30 feet from the toilet structure | | Yes=1, No= 0  (skip to ‘6.8’ if ‘0’) |  |
| 6.7 | Water and soap/ash/mud available within 30 feet from the toilet structure | | Yes=1, No= 0 |  |
| 6.8 | Mother’s hand appears clean | | Yes=1, No= 0 |  |
| 6.9 | Child’s hand appears clean | | Yes=1, No= 0 |  |
| **Safe water use** | | | | |

|  | Use | Source | | | Do you/household own the  water sources? | Container to preserve  water (multiple  answer)  (skip to next if code is  ‘6’) | | Is the container  covered?  Yes [1]; No [0] |  | |
| --- | --- | --- | --- | --- | --- | --- | --- | --- | --- | --- |
|  |  | Rainy season | Dry season | |  |  |  |  |  |  |
| 6.10 | Water for drinking |  |  | |  |  | |  |  |  |
| 6.11 | Water for cooking |  |  | |  |  | |  |  |  |
| 6.12 | Water for cleaning |  |  | |  |  | |  |  |  |
| 6.13 | Water for washing hand before serving food |  |  | |  |  | |  |  |  |
| 6.14 | Water for washing hand before eating food |  |  | |  |  | |  |  |  |
|  |  | Tube well [1]  River/canal/ pond/ditch [2] Pipe water [3]  Others [specify] [4]  Don’t wash hand [0] | | | Yes [1] No [2] Share [3] | Pitcher [1], Bucket [2] Jala/motka [3]  Drum [4]  Others (specify) [5] Don’t preserve [6] | |  |  |  |
| 6.15 | What is the distance of water source from the household? | | | Drinking water meter | | |  | | | |
|  |  |  |  | Other consumable water meter | | |  |  |  |  |
| 6.16 | Has the water source(s) been tested for arsenic (within past one year)? (applicable for tubewell only) | | | Yes [1]; No [2]; NA [99] | | |  | | | |
| 6.17 | Do you do anything to the water to make it safe for drinking? | | | Yes [1]; No [2]  (skip to ‘6.19’ if ‘0’) | | |  | | | |
| 6.18 | What do you usually do to make the water safe? | | | 1=Boil  2=Add bleaching/chlorine/use purify tablet 3=Strain through a cloth  4=Use water filter (ceramic/sand/composite etc.) 5=Let it stand and settle  6=Other(specify)  88=Don’t know | | |  | | |  |
| 6.19 | Household stored leftover food | | | Yes [1]; No [2]  (skip to ‘H7.1’ if ‘0’) | | |  | | | |
| 6.20 | Household stored leftover food that was kept covered | | | Yes [1]; No [2] ; NA [99] | | |  | | | |
| 6.21 | How much iron do you have in your tubewell water? | | | None [1]; Very little [2]; Some [3]; Lot [4]; NA [99] | | |  | | | |

**H7. Household dietary diversity score (HDDS)**

**Note: Ask the person responsible for food preparation. If the household had funeral, feast or most members were absent ask for data of an earlier day**

| ***I would like to ask you about the foods that members of your household consumed during the previous 24 hours:*** | | | | | Household consumed during  the previous 7 days  (specify number of days) |
| --- | --- | --- | --- | --- | --- |
| **Sl.** | **Food group** | **Examples** | **Yes=1  No=0** (skip to ‘7  days’ q. if ‘0’) | Source  1=Self produced, 2=Wild  catch/gathered, 3=Purchased,  4=Managed/charity |  |
| 7.1 | Cereals | corn, rice, wheat or any other grains or foods made from these (e.g. bread, noodles, shuzi or other grain products) + insert local foods |  |  |  |
| 7.2 | White roots and tubers | white potatoes, white yam, or other foods made from roots |  |  |  |
| 7.3 | Vitamin A rich vegetables and tubers | pumpkin, carrot or sweet potato that are orange inside + other locally available vitamin A rich vegetables (e.g. red sweet pepper) |  |  |  |
| 7.4 | Dark green leafy Vegetables | dark green leafy vegetables, including wild forms + locally available vitamin A rich leaves such as amaranth, spinach |  |  |  |
| 7.5 | Other vegetables | other vegetables (e.g. tomato, onion, eggplant) + other locally available vegetables |  |  |  |
| 7.6 | Vitamin A rich fruits | ripe mango, ripe papaya and 100% fruit juice made from these + other locally available vitamin A rich fruits |  |  |  |
| 7.7 | Other fruits | other fruits, including wild fruits and 100% fruit juice made from these |  |  |  |
| 7.8 | Organ meat | liver, kidney or other organ meats |  |  |  |
| 7.9 | Flesh meat | beef, lamb, goat, rabbit, game, chicken, duck, other birds |  |  |  |
| 7.10 | Eggs | eggs from chicken, duck, koel or any other egg |  |  |  |
| 7.11 | Fish and seafood | fresh fish small |  |  |  |
|  |  | fresh fish medium and large |  |  |  |
|  |  | dried fish |  |  |  |
|  |  | Shellfish |  |  |  |
| 7.12 | Legumes, nuts and seeds | dried beans, dried peas, lentils, nuts, seeds or foods made from these (eg. hummus, peanut butter) |  |  |  |
| 7.13 | Milk and milk products | milk, cheese, yogurt or other milk products |  |  |  |

| 7.14 | Oils and fats | oil, fats or butter added to food or used for cooking |  |  |  |
| --- | --- | --- | --- | --- | --- |
| 7.15 | Sweets | sugar, honey, sweetened soda or sweetened juice drinks, sugary foods such as chocolates, candies, cookies and cakes |  |  |  |
| 7.16 | Spices, condiments, beverages | spices (black pepper, salt), condiments (soy sauce, hot sauce), coffee, tea |  |  |  |
| ^7^.^17^ | Miscellaneous (drinks) | tea, bottled juice, coffee, cold drinks |  |  |  |

**H8. Food expenditure: monthly recall (last month)**

**Consultant’s Line number#**

| Insert the type of food | | Amount | Units for price  gram [1]  KG [2]  number [3] | What is the monetary value per  unit?  (Taka) |
| --- | --- | --- | --- | --- |
| 8.1 Have you purchased rice/wheat/bread in the last month? | | | | Yes=1, No=0  (skip to ‘8.2’ if ‘0’) |
| 8.1.1 | Rice |  |  | Tk |
| 8.1.2 | Wheat/Flour/*Suzi* |  |  | Tk |
| 8.1.3 | Baked Bread/Bread |  |  | Tk |
| 8.1.4 | Flattened rice/Puffed rice |  |  | Tk |
| 8.1.5 | Vermicelli |  |  | Tk |
| 8.1.6 | Biscuit |  |  | Tk |
| 8.2 Have you purchased pulse in the last month? | | | | Yes=1, No=0  (skip to ‘8.3’ if ‘0’) |
| 8.2.1 Pulse/lentil | | | | Tk |
| 8.3 Have you purchased oil in the last month? | | | | Yes=1, No=0  (skip to ‘8.4’ if ‘0’) |
| 8.3.1 | Oalm/Soybean oil |  |  | Tk |
| 8.3.2 | Mustard oil |  |  | Tk |
| 8.4 Have you purchased meat/fish the last month? | | | | Yes=1, No=0  (skip to ‘8.5’ if ‘0’) |
| 8.4.1 | Fish |  |  | Tk |
| 8.4.2 | Meat |  |  | Tk |
| 8.5 Have you purchased egg in the last month? | | | | Yes=1, No=0  (skip to ‘8.6’ if ‘0’) |
| 8.5.1 Egg | | | | Tk |
| 8.6 Have you purchased dairy products the last month? | | | | Yes=1, No=0  (skip to ‘8.7’ if ‘0’) |

| 8.6.1 | Milk |  |  | Tk |
| --- | --- | --- | --- | --- |
| 8.6.2 | Milk products |  |  | Tk |
| 8.7 Have you purchased vegetables/fruits in the last month? | | | | Yes=1, No=0  (skip to ‘8.8’ if ‘0’) |
| 8.7.1 | Leafy vegetable |  |  | Tk |
| 8.7.2 | Non leafy vegetables |  |  | Tk |
| 8.7.3 | Root vegetables |  |  | Tk |
| 8.7.4 | Fruits |  |  | Tk |
| 8.7.5 | Fruits- Banana only |  |  | Tk |
| 8.8 Have you purchased raw/powdered spices in the last month? | | | | Yes=1, No=0  (skip to ‘8.9’ if ‘0’) |
| 8.8.1 | Onion |  |  | Tk |
| 8.8.2 | Garlic |  |  | Tk |
| 8.8.3 | Ginger |  |  | Tk |
| 8.8.4 | Chilli/ dried chilli |  |  | Tk |
| 8.8.5 | Cumin/coriander |  |  | Tk |
| 8.9 Have you purchased any snacks/fast food in the last month? | | | | Yes=1, No=0  (skip to ’8.10’ if ‘0’) |
| 8.9.1 | Fast food e.g. potato chips/*puri/peyaju/cake/chanachur* |  |  | Tk |
| 8.10 Have you purchased any tea/sugar in the last month? | | | | Yes=1, No=0  (skip to ‘8.11’ if ‘0’) |
| 8.10.1 | Tea |  |  | Tk |
| 8.10.2 | Sugar/jaggery |  |  | Tk |
| 8.10.3 | Condensed Milk |  |  | Tk |
| 8.11 Have you purchased betel leaf in the last month? | | | | Yes=1, No=0  (skip to ‘8.12’ if ‘0’) |
| 8.11.1 Betel leaf/nut | | | | Tk |
| 8.12 Others | | | | Yes=1, No=0  (skip to ‘H9A.1’ if ‘0’) |
| 8.12.1 | Specify |  |  | Tk |
| 8.12.2 | Specify |  |  | Tk |

**H9: Non-food expenditure:**

**Consultant’s Line number#**

**H9. A Non-food expenditure: recall period last 1 month**

| **Item Name** |  | **Cash expenditure** | **If not spent in cash but consumed what  is the value of consumption, that you  received/gathered from other sources** | | |
| --- | --- | --- | --- | --- | --- |
| **FUEL AND LIGHTING** |  |  |  | | |
| 9A.1 Have you purchased any fuel and lighting materials in the last month?? (Firewood, Cow dung, etc.) |  | Yes =1, No=0 | |  |  |
|  | Tk. |  | Tk. | | |
| **COSMETICS AND OTHER EXPENSES** |  |  |  | |  |
| 9B.2 Have you purchased any cosmetics in the last month? (Soap, Shampoo, Toothpaste, Snow, Cream, Powder, Perfume, etc.) |  | Yes =1, |  |  |  |
|  |  |  | No =0 |  |  |
|  | Tk. |  | Tk. | | |
| **WASHING AND CLEANING EXPENSES** |  |  |  | | |
| 9B.3 Have you purchased any washing and cleaning materials in the last month? (*Ball-soap,* detergent, Ashes, etc) |  | Yes =1, No =0 | |  |  |
|  | Tk. |  | Tk. | | |
| **TRANSPORT/ TRAVEL AND MAINTENANCE** |  |  |  | | |
| 9B.4 Did you pay any fare for transport in the last month? (Bus fare, Rickshaw/van fare, CNG, Bicycle maintenance, etc.) |  | Yes =1, No=0 | |  |  |
|  | Tk. |  | Tk. | | |
| **OTHER MISC. CHARGES** |  |  |  | | |
| 9B.5 Did you pay any bill/salary in the last month? (Mobile recharge, electricity bill, etc.) |  | Yes =1, No=0 | |  |  |
|  | Tk. |  | Tk. | | |
|  |  |  |  | | |

**H9.B Non-food expenditure: recall period last 1 year**

| **Item Name** |  | **Cash expenditure** |  | **If not spent in cash but consumed what  is the value of consumption, that you  received/gathered from other sources** |
| --- | --- | --- | --- | --- |
| **GARMENT, CLOTHING MATERIALS, FOOTWARE, HOUSEHOLD TEXTILES** |  |  |  |  |
| 9B.1 Have you purchased any readymade garmenting the last 1 year?  (Lungi/dhuti, Shirts, Pant, Towel/gamcha, Cloth/ fabric, Tailoring expenses, Shoes, Bed sheets Curtain, etc.) |  |  | Yes =1,  (skip to ‘9B.2’ | No=0  if ‘0’) |
|  |  |  |  |  |
|  | Tk. |  | Tk. | |

| **HOUSING RELATED EXPENSES** |  | | | | | | | | | |
| --- | --- | --- | --- | --- | --- | --- | --- | --- | --- | --- |
| 9B.2 Did you pay for any rent/charges in last 1 year? (House rent, Disaster-related maintenance/ repair) | Yes =1, No =0  (skip to ‘9B.3’ if ‘0’) | |  | | | |  | | | |
|  | Tk. | Tk. | | | | | | | | |
| **MEDICAL TREATMENT EXPENSES** |  |  | | | | | | | | |
|  | Yes =1, No =0 | |  | | | |  | | | |
| 9B.3 Did you pay for any medical expenses in last 1 year? (Doctor's fees, Health-related travel/incidental expenses, etc.) |  |  |  |  |  |  |  |  |  |  |
|  | ‘9B.4’ if ‘0’)  (skip to | |  | | | |  | | | |
|  | Tk. | Tk. | | | | | | | | |
| **EDUCATIONAL EXPENSES** |  |  | | | | | | | | |
| 9B.4 Did you pay for any educational expenses in last 1 year? (Personal teaching expenses, Text book/ note books/ stationary, etc.) | Yes =1, No =0  (skip to ‘9B.5’ if ‘0’) | |  | | | | | | | |
|  |  |  |  | | | | | | | |
|  | Tk. | Tk. | | | | | | | | |
| **REMITTANCES, CEREMONIES, GIFTS, ETC.** |  |  | | | | | | | | |
| 9B.5 Did you pay for any remittances/ceremonies in the last six months? (Remittances to others living separately, Donation/ sadqa, etc.) | Yes =1, No =0  (skip to ‘9B.6’ if ‘0’) | |  | | | |  | | | |
|  | Tk. | Tk. | | | | | | | | |
| **RECREATION & LEISURE, ETC.** |  |  | | | | | | | | |
|  | Yes =1, No (skip to ‘9B.7’ | =0 | |  | | | |  | | |
| 9B.6 Did you pay for any recreational activities in last 1 year? (Cinema, TV satellite cable fees, etc) |  |  |  |  |  |  |  |  |  |  |
|  |  | ‘0’)  if | |  | | | | | | |
|  | Tk. | Tk. | | | | | | | | |
| **TAXES, INTEREST, FINES ETC.** |  |  | | | | | | | | |
|  | Yes =1, No (skip to ‘9B.8’ | =0 | |  | | | |  | | |
| 9B.7 Did you pay any tax/charges in the last 1 year? (Legal practitioner fees, ect.) |  |  |  |  |  |  |  |  |  |  |
|  |  | if ‘0’) | | | | | | | | |
|  | Tk. | Tk. | | | | | | | | |
|  |  |  |  |  |  |  |  |  |  |  |
| **COOKING EQUIPMENT** |  |  | | | | | | | | |
| 9B.8 Have you purchased any cooking equipment in the last 1 year? (Stove, plates and dishes etc.) | Yes =1, No =0  (skip to ‘9B.9’ if ‘0’) | | | |  | | | |  | |
|  | Tk. | Tk. | | | | | | | | |
| **PERSONAL ARTICLES** |  |  | | | | | | | | |
|  | Yes =1, No (skip to ‘9B.10’ | =0 | | | |  | | | |  |
| 9B.9 Have you purchased any personal article in the last 1 year? (Jewellery, wrist watch/ clock etc.) |  |  |  |  |  |  |  |  |  |  |
|  |  | if ‘0’) | | | |  | | | |  |
|  | Tk. | Tk. | | | | | | | | |
| **MISC. HOUSEHOLD DURABLE** |  |  | | | | | | | | |
|  | Yes =1, No (skip to ‘9B.11’ | =0 | | | |  | | | |  |
| 9B.10 Have you purchased any electrical device/other equipments in the last 1 year? (Television, Mobile phone, etc.) |  |  |  |  |  |  |  |  |  |  |
|  |  | if ‘0’) | | | |  | | | |  |
|  | Tk. | Tk. | | | | | | | | |
| **INSURANCE EXPENDITURE** |  |  | | | | | | | | |
| 9B.11 Did you pay for any insurance in the last 1 year? (Insurance, Shomobay shomity) | Yes =1, No =0  (skip to ‘9B.12’ if ‘0’) | | | | |  | | | |  |
|  | Tk. | Tk. | | | | | | | | |
| **EQUIPMENT USED FOR FARMING/FISHERIES** |  |  | | | | | | | | |

| 9B.12 Have you purchased any equipment in the last 1 year that was used for farming or fisheries? (Seeds, Fish fingerlings, Spade/plow/weeder/hoes, etc.) |  | Yes =1, No =0  (skip to ‘9B.13’ if ‘0’) | |
| --- | --- | --- | --- |
|  | Tk. |  | Tk. |
| 1. Seed |  |  |  |
| 1. Seedling |  |  |  |
| 1. Sampling |  |  |  |
| 1. Fingerling |  |  |  |
| 1. Fertilizer |  |  |  |
| 1. Lime |  |  |  |
| 1. Fish feed |  |  |  |
| 1. Poultry feed |  |  |  |
| 1. Cheeks |  |  |  |
| 1. Vaccine |  |  |  |
| 1. Spade/plow/weeder |  |  |  |
| 1. Fishing net/hook |  |  |  |
| 1. Fencing material |  |  |  |
| **OTHERS** |  |  |  |
| 9B.12 Have you purchased any cigarette/biri/jarda in last 1 year |  | Yes =1, No =0  (skip to ‘9B.14’ if ‘0’) | |
|  | Tk. | Tk. | |
| 9B.13 Others |  | Yes =1, No =0  (skip to ’H10.1’ if ‘0’) | |
|  | Tk. | Tk. | |

**H10. Savings**

**Consultant’s Line number#**

| 10.1 | Do you and/your spouse is a member of any *shomobay shomity*/community savings committee/bank/microfinance institution? (except for *Suchana*) | | | | | Yes [1]; No [0] |  |
| --- | --- | --- | --- | --- | --- | --- | --- |
| 10.2 | Do you have *Suchana* group savings membership? | | | | | Yes [1]; No [0] |  |
| 10.3 | Do you and/or your spouse have any savings? | | | | | Yes [1]; No [0]  (skip to ’11.1’ if ‘0’) |  |
|  | 10.3.1 | 10.3.2 | 10.3.3 | 10.3.4 | 10.3.5 | 10.3.6 | 10.3.7 |
|  | Savings at home/  Savings with  money guard  (Taka) | Bank/ post office (Taka  ) | NGOs  (Taka) | Microfinance Institutions  (Taka) | Suchana group  savings  (Taka) | Others  (Taka) | What is the intended use?  (code 1) |
|  |  |  |  |  |  |  |  |
| **Code 1:**  To buy household goods 1  To buy productive assets for agriculture 2  To buy other productive assets 3  To start / help business 4  To buy land / house 5  For education / training 6  For marriage/dowry 7  To build / repair house 8 | | To get loan 9  To lend to others 10  To prepare for difficult times/danger 11  To send someone abroad for a job 12  For the future of children 13  Medical or other emergency 14  Don’t know/no special reason 15  Other (specify) 16 | | | |  | |

**H11. Loans**

| 11.1 Do you and/or your spouse have any loans outstanding? Yes [1]; No [0].  **(**skip to **^4^**12.1’ if **^g^**0’**)** | | | | | | | | |  |
| --- | --- | --- | --- | --- | --- | --- | --- | --- | --- |
| 11.2 | 11.3 | 11.4 | 11.5 | 11.6 | 11.7 | 11.8 | Code 2 | |  |
| Loan  Number | From  whom?  (code 1) | What was the  loan mainly  used for?  (first 3 reasons)  (code 2) | Whether in  Cash?  In Cash [1]  In kind [2] | Loan Value  (Taka) | Whose  loan?  Self [1]  Spouse's [2] | Are you  repaying the  loan?  Partially [1]  No [0] | Business enterprise 1  To buy fertilizer 2  To buy seeds 3  To buy pesticides . 4  To buy irrigation equipment 5  To buy other agricultural implements 6  To buy water for irrigation 7  Costs of diesel/electricity for agriculture...8 Labor wages for agriculture . 9  Costs of hired machines/animals for  agriculture...10  To buy productive assets  for purposes other than agriculture 11  For lease of land for agriculture (cash only) 12  For lease of land used for purpose  other than agriculture(cash only) 13 | Purchase land 14  To purchase cow/goat 15  For medical treatment 16  To meet household consumption needs 17  Rent/purchase/improve housing 18  Educational expenses 19  Marriage expenditure 20  Dowry 21  Funeral 22  To lend out at higher interest 23  To go abroad 24  To repay other loan 25  Other (specify) 26 |  |
|  | 1 | 2 | 3 | 4 | 5 | 6 |  |  |  |
| 1 |  |  |  |  |  |  |  |  |  |
| 2 |  |  |  |  |  |  |  |  |  |
| 3 |  |  |  |  |  |  |  |  |  |
| 4 |  |  |  |  |  |  |  |  |  |
| 5 |  |  |  |  |  |  |  |  |  |
| **Code 1: Sources of borrowing:**  Bank [1];Money-lender [2]; Shop-keeper [3]; Relative [4]; Friend/Neighbour [5]; Grameen [6]; ASA [7];  TMSS [8]; RDRS [9]; Proshika [10];  Padakhep [11];  Swanirwar [12];  CNRS [13];  FIVDB [14]; Other [15] | | | | | | | | | |

**H12. Household food insecurity access scale (HFIAS)**

| **Occurrence Questions** | Response | If yes, how often did this happen? |
| --- | --- | --- |
| 1. In the past four weeks, did you worry that your household would not have enough food? | [0] No [1] Yes  (skip to 12.2 if ‘0’) | 1=Rarely  2=Sometimes  3=Often |
| 2. In the past four weeks, were you or any household member not able to eat the kinds of foods you preferred because of a lack of resources? | [0] No [1] Yes  (skip to 12.3 if ‘0’) | 1=Rarely  2=Sometimes  3=Often |
| 3. In the past four weeks, did you or any household member have to eat a limited variety of foods due to a lack of resources? | [0] No [1] Yes  (skip to 12.4 if ‘0’) | 1=Rarely  2=Sometimes  3=Often |
| 4. In the past four weeks, did you or any household member have to eat some foods that you really did not want to eat because of a lack of resources to obtain other types of food? | [0] No [1] Yes  (skip to 12.5 if ‘0’) | 1=Rarely  2=Sometimes  3=Often |
| 5. In the past four weeks, did you or any household member have to eat a smaller meal than you felt you needed because there was not enough food? | [0] No [1] Yes  (skip to 12.6 if ‘0’) | 1=Rarely  2=Sometimes  3=Often |
| 6. In the past four weeks, did you or any household member have to eat fewer meals in a day because there was not enough food? | [0] No [1] Yes  (skip to 12.7 if ‘0’) | 1=Rarely  2=Sometimes  3=Often |
| 7. In the past four weeks, was there ever no food to eat of any kind in your household because of lack of resources to get food? | [0] No [1] Yes  (skip to 12.8 if ‘0’) | 1=Rarely  2=Sometimes  3=Often |
| 8. In the past four weeks, did you or any household member go to sleep at night hungry because there was not enough food? | [0] No [1] Yes  (skip to 12.9 if ‘0’) | 1=Rarely  2=Sometimes  3=Often |
| 9. In the past four weeks, did you or any household member go a whole day and night without eating anything because there was not enough food? | [0] No [1] Yes  (skip to 13.A.1 if ‘0’) | 1=Rarely  2=Sometimes  3=Often |
| 1 = Rarely (once or twice in the past four weeks)  2 = Sometimes (three to ten times in the past four weeks)  3 = Often (more than ten times in the past four weeks) | | |

**H13. Months of adequate household food provisioning Not to be included**

**H14. Coping Strategies**

**H14.A. Coping with crisis in the last year**

|  | Events | Has the event of [ ] occurred within the last 12 months? Yes [1]  No [0] (skip to next if ‘0’) | How much money  did you spend or  lose? (Taka) | How did you cope with the increase of spending money? (code 1) | If you received money from anyone in any way in these situations, from whom? (code 2) |
| --- | --- | --- | --- | --- | --- |
|  |  | 1 | 2 | 3 | 4 |
| H14A.1 | House damaged seriously due to any natural disaster |  |  |  |  |
| H14A.2 | Crops lost due to any natural disaster |  |  |  |  |
| H14A.3 | Serious illness of income earning HH member |  |  |  |  |
| H14A.4 | Serious illness of non-income earning HH member |  |  |  |  |
| H14A.5 | Income earning household member passed away |  |  |  |  |
| H14A.6 | Non-Income earning household member passed away |  |  |  |  |
| H14A.7 | Marriage of household member |  |  |  |  |
| H14A.8 | Divorce |  |  |  |  |
| H14A.9 | Abandon/Separated |  |  |  |  |
| H14A.10 | Loss of income earning household member |  |  |  |  |
| H14A.11 | Loss of land |  |  |  |  |
| H14A.12 | Loss of livestock/poultry due to natural causes |  |  |  |  |
| H14A.13 | Poisoning/damaging livestock by others |  |  |  |  |
| H14A.14 | Legal Case/dispute |  |  |  |  |
| H14A.15 | Theft |  |  |  |  |
| H14A.16 | Mugging/robbery |  |  |  |  |
| H14A.17 | People restricting movement |  |  |  |  |
| H14A.18 | Domestic violence |  |  |  |  |
| H14A.19 | Breaking up from nucleus family |  |  |  |  |
| H14A.20 | Other (specify) |  |  |  |  |
| **Code 1:** Didn’t do anything [0]; Reduce Consumption Expenditure [1]; Use savings [2] ; Asset sale [3]; Sending child to other household [4]; Sending child (less than 14) to work [5]; Sending previously non-working adult HH member to work [6]; Begging [7]; Borrowing [8]; Sell Advance Labor [9]; Relief Aid [10]; Transfer from friend/ relative [11]; Selling Agriculture/pisciculture produces [12];  Others (specify) [13] | | | | | |
| **Code 2:** Bank [1];Money-lender [2]; Shop-keeper [3]; Relative [4]; Friend/Neighbour [5]; Grameen [6]; ASA [7]; TMSS [8]; RDRS [9]; Proshika [10]; Padakhep [11]; Swanirwar [12]; CNRS [13]; BRAC [14]; VARD [15]; Shimantik [16]; HEED [17]; FIVDB [18]; Other [19]; Didn’t take anything [0] | | | | | |

**H14.B. Do you use one of the resilient technology/option for livelihood mentioned below**

| Early plantation | Yes [1]; No [0] |  |
| --- | --- | --- |
| Cage aquaculture | Yes [1]; No [0] |  |
| Raised bed gardening | Yes [1]; No [0] |  |
| Water tolerance variety selection | Yes [1]; No [0] |  |
| Vertical gardening | Yes [1]; No [0] |  |
| Sag bag gardening | Yes [1]; No [0] |  |
| Alternative livelihood activities | Yes [1]; No [0] |  |
| Late Plantation | Yes [1]; No [0] |  |
| Others (please specify) | **.........................** |  |

**H14.C. Government safety net programs (last 1 year**

| H14B.1 | Did you or any HH member(s)received any grant/allowance/stipend from the government? | Yes [1]; No [0]  (skip to ’H.15. 1’ if ‘0’) |  | | | | | | | |
| --- | --- | --- | --- | --- | --- | --- | --- | --- | --- | --- |
|  |  |  |  | | |  | |  | | |
|  |  |  |  | | | | | | | |
|  |  | If yes, use (code 1)  *Multiple answer |  |  |  | |  | |  |  |
|  |  |  |  | | | | | | | |
| Code 1: Maternity allowance [1]; Employment generation program for the poor [2]; Vulnerable group development [3]; School feeding program [4]; Old age allowance [5]; Widow, deserted destitute women allowance [6]; Allowance for financially insolvent disabled [7]; Allowance for poor lactating mother [8]; Honorarium for freedom fighters [9]; Medical allowance for freedom fighters [10]; Ration for Shaheed family/freedom fighters [11]; Livelihood improvement for tea garden laborors [12]; Stipend for disabled students [13]; CMS [14]; VGF [15]; Test Relief (TR) food [16]; Gratuitous Relief (GR) food [17]; Food For Work (FFW) [18]; Work For Money (WFM) [19]; Micro-credit for women self-employment [20]; Others (specify) | | | | | | | | | | |

**H15. Agriculture/pisciculture (last 1 year cultivation)**

**H15.1 Has your household grown crops, raised poultry or involved in fish farming on land/water body under its own operation (owned, leased in, rented in) in the last 12 months?**

Yes [1] No [0]

| SN# | a.Code | b.Type of  land/water  body | c.Area of  land/water  body  (do not  include area  of bank of  the water  bodies) | 1 year | | | | | | | | | | | | | | |
| --- | --- | --- | --- | --- | --- | --- | --- | --- | --- | --- | --- | --- | --- | --- | --- | --- | --- | --- |
|  |  |  |  | d.How much do  you have  currently  (skip to ‘g’ if ‘0’) | | e.How  much did  you sell | | f.How much did you earn by selling | g.How much  did you  consume | | h.How portion did you keep for next season/4 months (if applicable) | | i.How much  did you give  away? | | j.How much did you  keep to feed  poultry/livestock/fish? | | k.How much/many  was wasted/died? | |
|  | Code 1 | Code 2 | Decimal | Kg | Unit | Kg | Unit | Taka | Kg | Unit | Kg | Unit | Kg | Unit | Kg | Unit | Kg | Unit |
|  |  |  |  |  |  |  |  |  |  |  |  |  |  |  |  |  |  |  |
|  |  |  |  |  |  |  |  |  |  |  |  |  |  |  |  |  |  |  |
|  |  |  |  |  |  |  |  |  |  |  |  |  |  |  |  |  |  |  |
|  |  |  |  |  |  |  |  |  |  |  |  |  |  |  |  |  |  |  |
|  |  |  |  |  |  |  |  |  |  |  |  |  |  |  |  |  |  |  |

| **Code 1** |  |  |  |  | **Code 2** |
| --- | --- | --- | --- | --- | --- |
| **Food crops** | **Oil Seeds** | **Vegetables** | Cauliflower [617] | **Fish** | Homestead land [1] |
| Rice [11] | Mustard [41] | Pumpkin [61] | Turnip [618] | Rui [701] | Arable / arable land [^2^] |
| Wheat [12] | Soybean [42] | Eggplant [62] | Radish [619] | Katol [702] | Pasture for cattle grazing [3] |
| Other food crops (Maize, barley, barley, etc.) [13] | Others (Sesame, Linseed, etc.) [43] | Patal [63]  Okra [64] | Cowpea [620] Aram/*Kacumukhi* [621] | Mrigel [703] Telapia [704] | Bush / *jangla* Land [4] Cultivable pool [5] |
|  | **Spice** | Ridge gourd [65] | Green banana [622] | Pangash [705] | Others’ land/pool [6] |
| **Fiber crops** | Chili [51] | Bitter gourd [^66^] | Betel leaf [623] | Sherputi [706] | Waste / fallow land [7] |
| Dhonche [21] | Onion [52] | Danta [67] | Other vegetables [624] | Puti [707] | Riverbeds / *haor* Land [^8^] |
| Jute [22] | Garlic [53] | Gourd [68] | Water spinach [625] | Mola [708] | Pond/pool bank [9] |
| Other Fibre (Cotton, etc.) [^23^] | Other (ginger, coriander,  turmeric) [54] | Papaya [69] Lemon [610] Potato[611] | Orange sweet potato [626] Arum *latiraja* [627]  Red spinach [628] | Other fish [709]  Silver carp [710] Other big fish [711] |  |
| **Pulses [31]** |  | Bean [612] | **Fruits** | **Poultry** |  |
|  |  | Carrot [613] | Yellow fruits [71] | Birds (chicken/duck) only [801] |  |
|  |  | Tomatoes [614] | Green fruits [72] | Birds (chicken/duck) and eggs [^802^] |  |
|  |  | Moringa [615] |  |  |  |
|  |  | Leafy vegetables [616] |  |  | |

**H16. Participation in Social Programs/NGO participation**

| 16.1 | Have you or any household member became a member of *Suchana* core group? | Yes [1]; No [0] (skip to ‘16.11’ if ‘0’) |  |
| --- | --- | --- | --- |
| 16.2 | Did you or any household member receive any training for being a member of *Suchana* core group? | Yes [1]; No [0] (skip to ‘16.6’ if ‘0’) |  |
| 16.3 | What training(s) have you or the household member received for being a member of *Suchana* core group?  (*multiple response) | Fish farming [1]  Livestock rearing [2]  Poultry rearing [3]  On coping strategies [4]  On farming [5]  On nutrition [6]  On water, sanitation and hygiene [7] On gender/nurturing connections [8] Others (specify) [9] On gardening [10] |  |
| 16.4 | How many training session(s) have you or the household member attended in last 6 months after becoming a member of *Suchana* core group? |  |  |
| 16.5 | Who conducted the training sessions? (*multiple response) | Government officer [1]  CNRS [2] RDRS [3] FIVDB [4] Save The Children International [5]  World Fish [6]  iDE [7]  Helen Keller International [8]  Others (specify) [9]  Don’t know [10] |  |
| 16.6 | Have you or any household member received any grant from *Suchana*? | Yes [1]; No [0] (skip to ‘16.9’ if ‘0’) |  |
| 16.7 | What grant(s) have you or the household member received from *Suchana*?  (*multiple response) | Agricultural equipment [1]  Pisciculture equipment [2]  Poultry [3]  Livestock [4]  Communication with other businessmen [5] Communication with government agencies [6] Communication with other NGOs [7]  Cash [8] |  |

|  |  | Others (specify) [9]  Vegetable production inputs [10] |  |
| --- | --- | --- | --- |
| 16.8 | When did you or the household member receive the grant(s) from Suchana (approximate) | 1 Months ago  2  3  4  5  6  7  8  9  10  (Input as per the codes of 15.7) |  |
| 16.9 | Have you or any household member received any coaching after getting the *Suchana* grant(s)? | Yes [1]; No [0] (skip to ‘16.11’ if ‘0’) |  |
| 16.10 | How many coaching sessions in last 6 months have you or the household  member attended after receiving the *Suchana* grant(s)? |  |  |
| 16.11 | In last 1 year, have you or any household member took part in any NGO activities (except for *Suchana*) | Yes [1]; No [0]  (skip to ’H17.1’ if ‘0’) |  |
|  |  |  |  |
| 16.12 | Name of the NGO(s)  (*multiple response) | Grameen [1]  ASA [2] CNRS [3] RDRS [4] Proshikha [5]  Podokhep [6]  Shonirvor [7]  FIVDB [8] TMSS [9] BRAC [10] HEED [11] Shimantik [12]  Vard [13]  Others (specify) [14] |  |
| 16.13 | Have you received any training(s) from the NGO(s)? | Yes [1]; No [0] (skip to ‘16.15’ if ‘0’) |  |
| 16.14 | What training(s) have you received from the NGO(s)? (*multiple response) | Fish farming [1]  Livestock rearing [2] Poultry rearing [3]  On coping strategies [4] |  |

|  |  | On farming [5]  On gardening [6]  Others (specify) [7] |  |
| --- | --- | --- | --- |
| 16.15 | Have you or any household member received any grant from the NGO(s) | Yes [1]; No [0] (skip to ’H17.1’ if ‘0’) |  |
| 16.16 | What grants have you or the household member received from the NGO(s)  (*multiple response) | Agricultural equipments [1]  Pisciculture equipments [2]  Poultry [3]  Livestock [4]  Gardening inputs [5]  Communication with other businessmen [6] Communication with government agencies [7] Communication with other NGOs [8] Cash/microfinance [9]  Others (specify) [10] |  |

**H17. Morbidity**

17.1 Has any household member became sick in the last 15 days? Yes[1]; No[0] (skip to ‘17.2’ if ‘0’)

| Line No. | a.Type of illness for last 15 days (code 1) *multiple response | b. What was the first treatment sought?  (code 2)  (skip to ‘d’ if opt.1 or 2) | c. If the first  treatment sought was of a HCP, how many days after onset of illness  (days) | d. Total duration of illness  (from onset to recovery)  (days)  Still sick [88] | e. Total expenditure for treatment | | | f. Did illness interfere with any income generating activity? (>10 yrs old)  Yes [1]  No [0]  Still sick [88]  (skip to next if ‘0’) | g. If yes, then for how many days? |
| --- | --- | --- | --- | --- | --- | --- | --- | --- | --- |
|  |  |  |  |  | Visit | Exam/Medicine/  Hostital fee | Travel |  |  |
|  |  |  |  |  |  |  |  |  |  |
|  |  |  |  |  |  |  |  |  |  |
|  |  |  |  |  |  |  |  |  |  |
|  |  |  |  |  |  |  |  |  |  |
|  |  |  |  |  |  |  |  |  |  |
|  |  |  |  |  |  |  |  |  |  |

17.2 Has any household member remained sick for the last 1 year? Yes [1]; No [0] (skip to ‘d’ if ‘0’)

Note: Please note the information of the first 3 diseases for each member according to the duration of the morbidity (longest to shortest)

| Line No. | a.Type of illness for last 1 year  (Code 1**)** | b. What treatment was sought?  (Code 2**)** | c. Total duration of morbidity  (year ) (month ) | d. Did any child below 5 years has died in  this household during 5 years  Yes [1]; No [0] |
| --- | --- | --- | --- | --- |
|  |  |  |  |  |
|  |  |  |  |  |
|  |  |  |  |  |
|  |  |  |  |  |
|  |  |  |  |  |
|  |  |  |  |  |

| **Code 1: Type of illness/symptoms** | | **Code 2: The first treatment sought** |
| --- | --- | --- |
| Fever [1] | Otitis/ hearing problem [12] | No treatment [1] |
| Pain/Ache [2] | Pregnancy related problem [13] | Tradition home treatment /Self treatment with OTC drugs [2] |
| Weakness [3] | Reproductive organ related problem [14] | Village doctor [3] |
| Cold/Cough [4] | Anemia [15] | Paramedics/PC/CHCP/FWV/CHW/SS/HA/MA [4] |
| Skin Rash [5] | Diabetes [16] | Allopathic medicine seller (when identify disease and give treatment [5] Qualified |
| Loose bowel movement [6] | Hypertension [17] | government/nongovernmental MBBS [6]; Pir/Fakir/Traditional healer [7] Kabiraz/Hakim |
| Drowsiness [7] | Mental illness [18] | [8] |
| Vomit [8] | Dental problem [19] | Homeopathic [9]  Other, [10] specify |
| Lack of appetite [9] | Asthma [20] |  |
| Insomnia [10] | Throat swelling [21] |  |
| Night-blindness / cataract [11] | Gastric [22] |  |
|  | Tumor [23] |  |
|  | Other, [24] specify |  |

**H 18.A. Access to facilities**

| Code | List of Facilities | Does any household member visit this facility usually (when ever needed)?  Yes [1]  No [2]  (skip to next if ‘0’) | What mode of transportation do you normally use to get to the closest facilities?  (multiple responses possible- list 3)  Foot ..1  Bicycle ..2  Rickshaw/Van ..3  Bullock cart ..4  Boat ..5  Engine boat ..6  Motorcycle ..7  Tempo/Baby taxi/Nosimon..8  Bus ..9  Train 10  Other 11 | | | Distance  (km) |
| --- | --- | --- | --- | --- | --- | --- |
|  |  |  |  |  |  |  |
| 18.1 | Health centre/hospital |  |  |  |  |  |
| 18.2 | Local shop/shops |  |  |  |  |  |
| 18.3 | Weekly/periodic bazaar |  |  |  |  |  |
| 18.4 | Bank |  |  |  |  |  |
| 18.5 | Market (to purchase food/commodities)  GPS: |  |  |  |  |  |
| 18.6 | Market (to sell food/commodities)  GPS: |  |  |  |  |  |
| 18.7 | Government Agricultural services (DAE) |  |  |  |  |  |
| 18.8 | Department of Fisheries (DoF) |  |  |  |  |  |
| 18.9 | Private sector- fish fingerling/feed, lime |  |  |  |  |  |
| 18.10 | Private sector- seedling, fertilizers |  |  |  |  |  |
| 18.11 | Public sector- fish fingerling/feed, lime |  |  |  |  |  |
| 18.12 | Public sector- seedling, fertilizers |  |  |  |  |  |

**H18.B. Quality inputs for Income Generating Activities (IGA)**

| Please mention whether you use the following inputs. Yes [1]; No [0]  (skip to next if ‘0’) | | What do you consider to ensure the inputs are of good quality?  * Multiple answers |
| --- | --- | --- |
| **a** | Seed | Date of expiration [1]  Date of packaging [2]  Batch number [3]  Name and reputation of the manufacturer/producer [4]  Name and reputation of the vendor [5]  Whether the packets/sacks are sealed properly [6]  Price [0]  Don’t know [44] |
| **b** | Fertilizer | Date of expiration [1]  Date of packaging [2]  Batch number [3]  Name and reputation of the manufacturer/producer [4]  Name and reputation of the vendor [5]  Whether the packets/sacks are sealed properly [6]  Composition label [7]  Note on direction of use [8]  Price [0]  Don’t know [44] |
| **c** | Fingerling | Size [1]  Health [2]  Origin i.e. name of hatchery or wild [3]  Name and reputation of the vendor [4]  Stocking mechanism i.e. how they were kept before selling [5]  Price [0]  Don’t know [44] |
| **d** | Lime | Date of expiration [1]  Date of packaging [2]  Batch number [3]  Name and reputation of the manufacturer/producer [4]  Name and reputation of the vendor [5]  Whether the packets/sacks are sealed properly [6]  Price [0]  Don’t know [44] |
| **e** | Cheek | Size [1]  Health [2]  Origin [3]  Name and reputation of the vendor [4]  Stocking mechanism i.e. how they were kept before selling [5]  Price [0]  Don’t know [44] |
| **f** | Vaccine | Date of expiration [1]  Date of packaging [2]  Batch number [3]  Name of the manufacturer [4]  Name and reputation of the vendor [5]  Whether the packets are sealed properly [6]  Price [0]  Don’t know [44] |
| **g** | Poultry feed | Date of expiration [1]  Date of packaging [2]  Batch number [3]  Name and reputation of the manufacturer/producer [4]  Name and reputation of the vendor [5]  Whether the packets/sacks are sealed properly [6]  Composition label [7]  Note on direction of use [8]  Price [0]  Don’t know [44] |
| **h** | Fish feed | Date of expiration [1]  Date of packaging [2]  Batch number [3]  Name and reputation of the manufacturer/producer [4]  Name and reputation of the vendor [5]  Whether the packets/sacks are sealed properly [6]  Composition label [7]  Note on direction of use [8]  Price [0]  Don’t know [44] |
| **i** | Sapling | Size [1]  Health [2]  Name and reputation of the nursery [4]  Name and reputation of the vendor [5]  Price [0]  Don’t know [44] |
| **j** | Seedling | Size [1]  Health [2]  Name and reputation of the nursery [4]  Name and reputation of the vendor [5]  Price [0]  Don’t know [44] |

**M1. Reproductive history of the mother**

|  | How old were you during your first marriage? | ………… year  Don’t know [44] |  |
| --- | --- | --- | --- |
|  | What was your age at that time of your first pregnancy? | ………… year  Don’t know [44] |  |
|  | How many times have you been pregnant?  (total number of times including abortion/MR and this pregnancy) | ……………Times |  |
|  | Results of conception(s) | Live birth ……………Times  Abortion ……………Times  Menstrual regulation (MR) ……………Times  Still birth ……………Times  Intrauterine death (IUD)……………Times |  |
|  | How many live birth children of yours has died and at what age? | No one died [88]  Neonate ……………Person  Within 1 year ……………Person  Within 2 years ……………Person  Within 5 years ……………Person  Others (specify) ……………Person  **Total ……………Person**. |  |
|  | Did you consume iron and folic acid during the last pregnancy? | Yes [1]; No [2]; Don’t know [44]  (skip to 1.9 if ‘0’) |  |
|  | If you did consume iron and folic acid during the last pregnancy then when did you start? | ……………… Month |  |
|  | On average, how many iron and folic acid did you consume per month during last pregnancy? | 1^st^ trimester ……………  2^nd^ trimester ……………  3^rd^ trimester …………… |  |
|  | On average, how many times did you go for health check-ups during your pregnancy for youngest child? **(ANC)** | ……………Times |  |
|  | Where did you go for health check-ups before delivery during your last pregnancy?  *Multiple response | UpaZila/Government medical Health Complex [1]  Zila hospital [2]  Maternity center [3]  Family planning center (FWC/CC) [4]  BRAC shushastho [5]  Private hospital/clinic [6]  Private chamber [7]  Local NGO health center [8]  *Kobiraj*/village doctor [9]  Pharmacy [10]  At home [11]  Don’t know [12]  Other (specify) [13] ……………… |  |
|  | To whom did you go for health check-ups before delivery during your last pregnancy? | Village doctor [1]  BRAC *shasthoshebika* [2]  Family Welfare Visitor (FWV) [3]  Female Welfare Assistant (FWA) [4]  Traditional Birth Attendant (TBA) [5]  Skilled Birth Attendant (SCBA) [6]  Homeopath doctor [7]  *Kobiraj* [8]  *Pir Fakir*/traditional healer [9]  MBBS doctor [10]  *Pushti apa* (CNO/CNP) [11]  Pharmacist [12]  Nurse/Paramedic [13]  Other (specify) [14] ………… |  |
|  | Who delivered your last baby? | Family Welfare Visitor (FWV/HI) [1]  Family Welfare Assistant (FWA/CHCP/HA) [2]  Traditional Birth Attendant (TBA) [3]  Skilled Birth Attendant (SCBA) [4]  BRAC *shasthoshebika* [5]  Nurse/Paramedic [6]  Relative/Friend/Neighbour [7]  MBBS doctor [8]  Self/No one [9]  Other (specify) [10] ………… |  |
|  | How was the delivery completed during your last pregnancy | Normal [1]  Episiotomy [2]  Forep/Vaccum [3]  Caesarean [4] |  |
|  | Where was the baby delivered? | At home [1]  On the way [2]  Zila hospital [3]  UpaZila/Government medical Health Complex [4]  Maternity center (MCWC) [5]  Family planning center (FWC) [6]  BRAC shushastho [7]  Private hospital/clinic [8]  Local NGO health center [9]  Other (specify) [10] ………… |  |
|  | As for the last child, did you receive any post natal care **(PNC)**? | Yes [1]; No [0]  **এখনও সময় আছে [3]**  (skip to ‘1.18’ if ‘0’) |  |
|  | How many times did you receive PNC | ……………Times |  |
|  | How many days after the delivery did you receive PNC | …………… Days |  |
|  | Have you received Vitamin A capsule after the last delivery? (within 45 days after delivery) | Yes [1]; No [0]; still have time [3] |  |
|  | Did you receive any tetanus vaccination (TT) during pregnancy? | Yes [1]; No [0]; NA [99] |  |
|  | During the last pregnancy did you eat more, the same, or less than you did before you were pregnant? | Less [1]; Same [2]; More [3] |  |
|  | During the last pregnancy did you rest more, the same, or less during day time than you did before you were pregnant? | Less [1]; Same [2]; More [3] |  |

**M2. Maternal knowledge**

**M2.1. Health, nutrion knowledge and general health awareness**

|  | Mother’s line number from HH section | #**...........................** |  |
| --- | --- | --- | --- |
|  | Did you hear about consuming Vitamin A capsule after child delivery? | Yes [1]; No [0]  (skip to ‘2.1.4’ if ‘0’) |  |
|  | What are the importances of consuming Vitamin A capsule after child delivery?  *Multiple answer | Prevents night blindness [1]  Prevents diseases [2]  Other (specify) [3] …………  Don’t know [4]  NA [99] |  |
|  | Did you hear about children consuming Vitamin A capsule? | Yes [1]; No [0] |  |
|  | Have you ever heard of iron intake during pregnancy? | Yes [1]; No [0]  (skip to ‘2.1.8’ if ‘0’) |  |
|  | How many iron tablets should you consume during pregnancy? | ……………………..; Don’t know [44] |  |
|  | What are the benefits of taking iron during pregnancy?  *Multiple answer | Prevents mother from anaemia [1]  Prevents child from anaemia at birth and later ages [2]  Develops brain of the child in the uterus [3]  Other (specify) [4] …………  Don’t know [44] |  |
|  | In your opinion, what causes anaemia?  *Multiple answer | Deficiency of iron (iron rich food) [1]  Worm infestation [2]  Other (specify) [3] …………  Don’t know [4] |  |
|  | What should children consume during diarrhea? | Packed ORS [1]  Homemade ORS [2]  Zinc syrup/tablets [3]  Rice powder [4]  Normal/regular food [5]  Other medicine [6] |  |
|  | For a women/girl, what should be the minimum age for marriage? | ……………Years; Don’t know [99] |  |
|  | What are the consequences of early marriage?  *Multiple answer | Discontinue of education [1]  Increases vulnerability to health problems [2]  Barriers to accessing employment [3]  Gender inequality [4]  Premature pregnancy [5]  Less power/voice in her new family [6]  Other (specify) [7] …………  Don’t know [44] |  |
|  | What are the consequences of early pregnancy?  *Multiple answer | Low birth weight baby [1]  Preterm birth [2]  Pregnancy induced hypertension [3]  Anemia [4]  Urinary tract infection [5]  Abnormal labor or caesarean section [6]  Other (specify) [7] …………  Don’t know [44] |  |
|  | To whom should you go for health check-ups during pregnancy?  *Multiple answer | Village doctor [1]  BRAC *shasthoshebika* [2]  Family Welfare Visitor (FWV) [3]  Female Welfare Assistant (FWA) [4]  Traditional Birth Attendant (TBA) [5]  Skilled Birth Attendant (SCBA) [6]  Homeopath doctor [7]  *Kobiraj* [8]  *Pir Fakir*/traditional healer [9]  MBBS doctor [10]  *Pushti apa* (CNO/CNP) [11]  Pharmacist [12]  Nurse/Paramedic [13]  Other (specify) [14] ………… |  |
|  | At least how many times you should go for health check-ups during pregnancy? | ………………………….Times; Don’t know [44] |  |
|  | How much you should sleep in day time during pregnancy in comparison to usual time? | Less [1]; Same [2]; More [3] |  |
|  | How much you should eat in during pregnancy in comparison to usual time? | Less [1]; Same [2]; More [3] |  |
|  | What type of salt do you use? (to confirm, please see the packet if you can) | Crude salt [1]; Packed salt [2]; Iodized salt [3]; Open salt [4]; Open but iodized salt [5] |  |

**M2.2. Knowledge on health services**

Read: Now I would like to ask you some questions regarding the information related to health services present in your community

| 2.2.1 | Do you know from where you can get general treatment in your community?  *(Insert first 3 options)* | Field health worker [1]  Health and family planning centre [2]  UpaZila/Government medical hospital [3]  Medical college hospital [4]  District hospital [5]  Local NGO [6]  Private clinic [7]  Drug shop [8]  MBBS doctor [9]  Panel doctor [10]  Kobiraz/village doctor [11]  homeopathy [12]  Community clinic [13]  Others, specify [14]……………….  Don’t know [44] |  |
| --- | --- | --- | --- |
| 2.2.2 | How do you get this information?  *Multiple answer | Local NGO staff [1]  Know from the beginning [2]  Other health worker [3]  Neighbor/ friends [4]  Relatives [5]  Radio [6]  TV [7]  Print media [8]  Posters [9]  Others, specify [10] ………… |  |

**M2.3. Knowledge on infant feeding practices**

|  | Immediately after birth what needs to be fed to the baby?  *(Only one answer)* | 1=Colostrums  2=Breast milk (without colostrums)  3=Other milk  4=Water  5=Honey/Sugar water  6=Other (Specify)  44=Don’t know |  |
| --- | --- | --- | --- |
|  | When breastfeeding should be initiated after birth? | Immediately  Hours  Days |  |
|  | Do you know till what age you should exclusively breastfed your child? | ……………… Days |  |
|  | Till what age should you breastfeed the baby? | ________________Years; Don’t know [44] |  |
|  | When should semi-solid food be introduced to the baby? | ________________ Months; Don’t know [44] |  |
|  | What food should you give to children aged between 6-23 months?  *Multiple answer | Bread, rice, biscuits, or any other foods made from cereals [1]  Potatoes, yams or any other foods made from roots or tubers [2]  Dried beans, dried peas, lentils or foods made from these [3]  Nuts, seeds or foods made from these [4]  Milk, cheese, yogurt or other milk products [5]  Any meat including organ meat [6]  Any fish [7]  Eggs [8]  Vitamin A rich fruit or vegetable like mango, papya, carrot, sweet potato [9]  Other fruits and vegetables [10] |  |
|  | What should you feed your baby (according to his/her age)? | Only breast milk [1]  Breast milk and semi-solid food [2]  All types of food [3]  Fruits and vegetables [4]  Food containing protein [5]  Others (specify) [6] |  |
|  | How many times should you feed your baby (according to his/her age) everday? | ________________Times; Don’t know [44] |  |
|  | How do you treat children during diarrheal episodes?  *Multiple answer | Packed ORS [1]  Homemade ORS [2]  Zinc syrup/tablets [3]  Rice powder [4]  Normal/regular food [5]  Other medicine [6]  Don’t know [44] |  |

**M2.4. Knowledge and practice regarding hand washing**

|  | When should you wash your hands? (*do not read the options,probe the mother by saying ‘anything else’*)  (multiple answers possible) | Before eating [1]  After using the toilet [2]  Before feeding the child [3]  After cleaning a child who has defecated [4]  Before preparing food [5]  After touching domestic animals and birds and cleaning their waste [6]  Other (specify) [7] ………………….  Before serving food [8]  Don’t know [44] |  |
| --- | --- | --- | --- |
|  | Practice of hand washing: | | |
|  | \| Activities \| Do you wash your hands ……..?  Yes=1  No= 0 \| B. How do you wash your hands …….?  Soap and water=01, Ash and water=02,=Mud and water=03, only water=04, Others=77 \| \| --- \| --- \| --- \| \| a.Before cooking? \|  \| If Others (77), specify)……………………….. \| \| b.Before feeding the child? \|  \| If Others (77), specify)……………………….. \| \| c.Before taking food? \|  \| If Others (77), specify)……………………….. \| \| d.After cleaning baby’s bottom? \|  \| If Others (77), specify)……………………….. \| \| e.After defecation? \|  \| If Others (77), specify)……………………….. \| \| f.After touching domestic animals and birds and cleaning their waste? \|  \| If Others (77), specify)……………………….. \| \| g. Before serving food? \|  \| If Others (77), specify)……………………….. \| | | |

**M3. Access to nutrition services and health care**

|  | Where do you usually go when you are sick? | Community Clinic [1]  NGO run clinic [2]  Union Health complex [3]  UpaZila/Government medical Health Complex [4]  District Health Complex [5]  Private clinic [6]  Pharmacy/drug dispenser [7]  *Kobiraj*/Village doctor [8]  Hoemopath [9]  Others (specify) [10] …………………. |  |
| --- | --- | --- | --- |
|  | Do you attend courtyard sessions during EPI sessions in your community? | Yes [1]; No [0]; We do not have EPI sessions [3] |  |
|  | How often do the government health workers come to visit you? | Once a week [1]  Twice a month [2]  Once a month [3]  Once in every few months [4]  Does not come [5] |  |
|  | Do you get visit from NGO health professionals e.g. BRAC Shasthya Shebika? | Yes [1]; No [0] |  |
|  | Have you ever received any health or family planning service from Community Clinic? | Yes [1]; No [0]  (skip to ‘3.7’ if ‘0’) |  |
|  | How often do you go to Community Clinics? | Don’t know [1]  Once a week [2]  Twice a month [3]  Once a month [4]  Once in every few months [5] |  |
|  | Are you/your husband currently using any contraception method? | Yes [1]; No [0]  (skip to ‘M4.1’ if ‘0’) |  |
|  | Which method do you/your husband use? | Condoms [1]  Birth control pills [2]  Implant/Copper T [3]  Injectable contraception [4]  Vesectomy [5]  Tubectomy [6]  Traditional/safe period [7]  Others (specify) [8] …………………………. |  |

**M4. Women’s empowerment**

**M4.1. Participation in decision making/decision making ability**

| Major family purchases | Did you yourself participate in [ACTIVITY] decision making?  **Code:**  Yes [1]; No [0] | When decisions are made regarding [ACTIVITY], who is it that normally takes the decision?  **Code:**  Self [1]; Spouse [2]; Other HH members [3]; Other non-HH members [4]; Combined [husband/wife] [5] | How much input did you have in the decision?  **Code:**  No input [1]; Input in few decisions [2]; Input into some decisions [3]; Input into most or all decisions [4] |
| --- | --- | --- | --- |
| a.Livestock (e.g. cow, goat, etc.) |  |  |  |
| b.Poultry for rearing |  |  |  |
| c.Fish/fingerlings for rearing |  |  |  |
| d.Productive capital/business assets (e.g. rickshaw, boat, plough, etc.) |  |  |  |
| e.Land |  |  |  |
| f.Large non-productive/business assets (e.g. TV, chair, electric fan, etc.) |  |  |  |
| g.Kitchenware/utensils (e.g. pots, pan, jug, etc.) |  |  |  |
| **Food item and grocery shopping** |  |  |  |
| **Cooking/food preparation** |  |  |  |
| **Own health care and medical treatment** |  |  |  |
| **Child’s health care and medical treatment** |  |  |  |
| **Visiting relatives or paternal house** |  |  |  |

**M4.2. Respect and domestic violence**

|  | Question | **Response code** | Response Code |
| --- | --- | --- | --- |
| 4.2.1 | Are you satisfied your male relatives respect the contribution you make to your household? | Yes [1]; No [0]; Don’t know [3] |  |
| 4.2.2 | Are you satisfied other community members respect the contribution you make to your household? | Yes [1]; No [0]; Don’t know [3] |  |
| 4.2.3 | Do you get some or full assistance from the other household members for the following activities? (1 month recall) |  |  |
|  | 1. Cooking | Yes [1]; No [0] |  |
|  | 2. Gather water/firewood for the house | Yes [1]; No [0] |  |
|  | 3. Cleaning | Yes [1]; No [0] |  |
|  | 4. Child care | Yes [1]; No [0] |  |
|  | 5. Agricultural activities | Yes [1]; No [0] |  |
|  | 6. Going to market | Yes [1]; No [0] |  |
|  | 7. Selling produce | Yes [1]; No [0] |  |
|  | 8. Homestead gardening | Yes [1]; No [0] |  |
|  | 9. Homestead poultry rearing | Yes [1]; No [0] |  |
| 4.2.4 | Has any of the following happened to you in the past year? | Yes, often [1]  Yes, sometimes [2]  No [0] |  |
|  | Your husband threatened you with divorce? |  |  |
|  | Your husband threatened you with taking another wife? |  |  |
|  | Your husband, another family member, or household resident verbally abused you? |  |  |
|  | Your husband, another family member, or household resident physically abuse you? |  |  |

**M5. Women Dietary Diversity**

| Now I would like to ask you about the types of foods that you ate yesterday during the day and at night. | | **Coding categories** | |
| --- | --- | --- | --- |
| *Read the list of foods. Place a 1 in the box if anyone in the box if anyone in the household date the food in question, place a 0 in the box if no one in the household ate the food.* | | Type | Times |
| A | Any [any local foods], bread, rice, biscuits, or any other foods made from rice, wheat, or [any locally available grain] | A | \|___\| |
| B | Any potatoes, yams or any other foods made from roots or tubers? | B | \|___\| |
| C | Any dried beans, dried peas, lentils or foods made from these? | C |  |
| D | Any nuts, seeds or foods made from these | D |  |
| E | Any milk, cheese, yogurt or other milk products? | E |  |
| F | Any beef, lamb, goat, pork, chicken, duck, rabbit, wild game or other birds, liver, kidney, heart, or other organ meats? | F |  |
| G | Any fresh fish (small)? | G1.................................................... |  |
|  | Any fresh fish (large)? | G2..................................................... |  |
|  | Any dried fish ? | G3 |  |
|  | Any shellfish? | G4 |  |
| H | Any eggs? | H |  |
| I | Any Vitamin A rich fruit or vegetable like mango or papya? | I |  |
| J | Any Vitamin A rich vegetable like carrot or sweet potato? |  |  |
| K | Any dark green leafy vegetables? | J | \|___\| |
| L | Other vegetables? | K | \|___\| |
| M | Other fruits? | L | \|___\| |
| N | Any foods made with oil, fat, or ghee? | M | \|___\| |
| O | Any sugar or honey? | N | \|___\| |
| P | Any other foods, such as condiments, tea? | O | \|___\| |

**C1. Child feeding practices**

| 1.1 | Child line # from HH section |  |  |  |  |
| --- | --- | --- | --- | --- | --- |
| 1.2 | Mother line # from HH section |  |  |  |  |
| 1.3 | What is the name of your child? | (indexed child) |  | Name: |  |
| 1.4 | Child sex | Male [1], Female [2] |  |  |  |
| 1.5 | Child date of birth (date/month/year) |  |  |  | / |
| 1.6 | Child birth order |  |  |  |  |
| 1.7 | Birth weight | *Kg*  Don’t know [44] |  |  |  |
| 1.8 | Did you ever breastfeed ***(NAME)***? | Yes [1]; No [0] |  |  |  |
| 1.9 | How long after birth did you first put (name) to the breast?  *(If immediately, insert 1, if within 24 hour, insert the exact hour, if more than a day, insert exact number of day).* |  | Immediately [77]  Hours  Days |  |  |
| 1.10 | Did you give colostrum to this child? | Yes [1]; No [0] |  |  |  |
| 1.11 | Did you put anything in (child’s name) mouth before first breast milk (e.g. sugar or honey)? | Yes [1]; No [0]; Don’t know [44] |  |  |  |
| 1.12 | Did you put anything in (name’s) mouth within 3 days after birth? | Yes [1]; No [0]; Don’t know [44] | 1.12 |  |  |
| 1.13 | Did you put any food or drink in (name’s) mouth within 6 months after birth? (probe for water) | Yes [1]; No [0]; Don’t know [44]  (skip if age <6m) | 1.13 |  |  |

| 1.14 | At what age did you first put anything (food/drink) in (name’s) mouth outside breast milk | Days: Months: Haven’t |  | | yet [44] |  |
| --- | --- | --- | --- | --- | --- | --- |
|  |  |  |  | |  |  |
|  |  |  | given anything | |  |  |
| 1.15 | For how many days/months did you breast-feed the baby? | Days: Months: |  | |  |  |
|  |  |  |  | |  |  |
| 1.16 | Is the child still breastfed? | Yes [1]; No [0]  (skip to ‘1.19’ if ‘1’) | | | |  |
| 1.17 | If no, at what age did you stop breastfeeding? (Days) | Days: | | | |  |
| 1.18 | For how long did you breastfed the child? | Days: Months: |  | |  |  |
|  |  |  |  | |  |  |
| 1.19 | Was (NAME) breastfed yesterday during the day or night? | Yes [1]; No [0]; Don’t know [44] | | | |  |
| *Sometimes babies are fed breast milk in different ways, for example by spoon, cup or bottle. This can happen when the mother cannot always be with her baby. Sometimes babies are breastfed by another woman, or given breast milk from another woman by spoon, cup or bottle or some other way. This can happen if a mother cannot breastfeed her own baby.* | | | | | | |
| 1.20 | Did (NAME) consume breast milk in any of these ways yesterday during the day or at night? | Yes [1]; No [0]; Don’t know [44] | | | |  |
| 1.21 | Did (name) have had any of the following liquids/drink yesterday during the day or night? |  | Yes | No | Don’t know | Times_____ _ |
|  | A Plain water? | A | 1 | 0 | 88 |  |
|  | B Infant formula such as [insert local examples]? | B | 1 | 0 | 88 |  |
|  | C Milk such as tinned, powdered, or fresh animal milk? | C | 1 | 0 | 88 |  |
|  | D Juice or juice drinks? | D | 1 | 0 | 88 |  |
|  | E Clear broth? | E | 1 | 0 | 88 |  |
|  | F Yogurt? | F | 1 | 0 | 88 |  |
|  | G Thin porridge? | G | 1 | 0 | 88 |  |
|  | H Any other liquids such as [list other water- based liquids available in the local  setting]? | H | 1 | 0 | 88 |  |
|  | I Any other liquids? | I | 1 | 0 | 88 |  |

| 1.22 | Was (NAME) given [LOCAL NAME FOR ORS] yesterday during the day or at night? | | Yes [1]; No [0]; Don’t know [44] | |  |
| --- | --- | --- | --- | --- | --- |
| 1.23 | Was (NAME) given any vitamin drops or other medicines as drops yesterday during the day or at night? | | Yes [1]; No [0]; Don’t know [44] | |  |
| 1.24 | Did *(NAME)* eat any solid, semi foods yesterday during the day or at night? *IF ‘YES’ PROBE:* What kind of solid, semi-solid, or soft foods did *(NAME)* eat? | | Yes [1]; No [0]; Don’t know [44]  [skip to 1.29 if ‘0’] | |  |
| 1.25 | How many times did *(NAME)* eat any solid, semi foods yesterday during the day or at night? | | Number of times | |  |
| 1.26 | *Read: Now I would like to ask you about any foods or liquids that (INFANT NAME) ate in the last 24 hours. Yesterday during the day or night, did [name of the child] eat anything at home or outside home.* | | | | |
|  | A | Porridge, bread, rice, noodles or other foods made from grains | | Yes [1]; No [0]; Don’t know [44] | Times_____ |
|  | B | Pumpkin, carrots, squash or sweet potatoes that are yellow or orange inside | | Yes [1]; No [0]; Don’t know [44] |  |
|  | C | White potatoes, white yams, manioc, cassava or any other foods made from roots | | Yes [1]; No [0]; Don’t know [44] |  |
|  | D | Any dark green leafy vegetables | | Yes [1]; No [0]; Don’t know [44] |  |
|  | E | Ripe mangoes, ripe papayas or (insert other local vitamin A-rich fruits) | | Yes [1]; No [0]; Don’t know [44] |  |
|  | F | Any other fruits or vegetables | | Yes [1]; No [0]; Don’t know [44] |  |
|  | G | Liver, kidney, heart, or other organ meats | | Yes [1]; No [0]; Don’t know [44] |  |
|  | H | Any meat, such as beef, pork, lamb, goat, chicken, or duck | | Yes [1]; No [0]; Don’t know [44] |  |
|  | I | Eggs | | Yes [1]; No [0]; Don’t know [44] |  |
|  | J | Fresh or dried fish, shellfish, or seafood | | Yes [1]; No [0]; Don’t know [44] |  |
|  | K | Any foods made from beans, peas, lentils, nuts, or seeds | | Yes [1]; No [0]; Don’t know [44] |  |
|  | L | Cheese, yogurt, or other milk products | | Yes [1]; No [0]; Don’t know [44] |  |
|  | M | Any oil, fats, or butter, or foods made with any of these | | Yes [1]; No [0]; Don’t know [44] |  |
|  | N | Any sugary foods such as chocolates, sweets, candies, pastries, cakes, or biscuits | | Yes [1]; No [0]; Don’t know [44] |  |
|  | O | Condiments for flavor, such as chillies, spices, herbs, or fish powder | | Yes [1]; No [0]; Don’t know [44] |  |
|  | Q | Snacks or fast food, such as chips | | Yes [1]; No [0]; Don’t know [44] |  |
|  | R | Miscellaneous: Tea, soft drinks/ juice | | Yes [1]; No [0]; Don’t know [44] |  |

| 1.27 | Did ***(NAME)*** drink anything from a bottle with a nipple yesterday during the day or night? | Yes [1]; No [0]; Don’t know [44] |  |
| --- | --- | --- | --- |
| *Now I would like to ask you about some particular foods (NAME) may eat. I am interested in whether your child had the item even if it was combined with other foods. Yesterday, during the day or night, did (NAME) consume any [list iron fortified solid, semi- solid or soft foods designed specifically for infants and young children available in the local setting]?* | | | |
| 1.28 | Have you ever heard about powder/sprinkles that can be added to food (use names of locally available products)? | Yes [1]; No [0] |  |
| 1.29 | Yesterday, during the day or night, did (NAME) consume any food to which you added powder or sprinkles? | Yes [1]; No [0] |  |
| 1.30 | Did anybody ever talk to you about how to feed your child? | Yes [1]; No [0]  (skip to ‘1.36’ if ‘0’) |  |
| 1.31 | If yes who? *Multiple answer | Health worker [1]  NGO worker [2]  Mother/Mother in law/Family member [3] Neighbors [4]  Other (specify) [5] |  |
| 1.32 | Did you hear/see/read any messages about breast feeding or complementary feeding? | Yes [1]; No [0]; Don’t know [44]  (skip to ‘1.38’ if ‘0’ or ‘88’) |  |
| 1.33 | Where or from whom did you hear /see/ read about it? | Radio [1]  TV [2]  Newspaper [3]  Doctor/HW/Shebika [4] Poster/leaflet/billboard [5]  Neighbors [6]  Mobile phone [7]  Home visits by HW [8]  Community [9]  Family members [10]  Suchana staff [11]  Clinics/EPI/Courtyard sessions [12] Others (Specify) [13] |  |
| 1.34 | Do you usually (once a week) listen to the radio and watch television (even in someone else’s place) | Yes [1]; No [0] |  |
| 1.35 | Did the child receive following vaccines through EPI at any time? | Have received and have card [1]  Have received but do not have card [2]  Did not receive [3]  Skip to 1.36 if ‘2’ or ‘3’ | |

|  |  | BCG (1^st^ dose) | 01=Yes, 02= No, 88 = Don’t know |  |  |
| --- | --- | --- | --- | --- | --- |
|  |  | BCG (2^nd^ dose) | 01=Yes, 02= No, 88 = Don’t know |  |  |
|  |  | Penta (1^st^ dose) | 01=Yes, 02= No, 88 = Don’t know |  |  |
|  |  | Penta (2^nd^ dose) | 01=Yes, 02= No, 88 = Don’t know |  |  |
|  |  | Penta (3^rd^ dose) | 01=Yes, 02= No, 88 = Don’t know |  |  |
|  |  | OPV (1^st^ dose) | 01=Yes, 02= No, 88 = Don’t know |  |  |
|  |  | OPV (2^nd^ dose) | 01=Yes, 02= No, 88 = Don’t know |  |  |
|  |  | OPV (3^rd^ dose) | 01=Yes, 02= No, 88= Don’t know |  |  |
|  |  | PCV (1^st^ dose) | 01=Yes, 02= No, 88= Don’t know |  |  |
|  |  | PCV (2^nd^ dose) | 01=Yes, 02= No, 88= Don’t know |  |  |
|  |  | PCV (3^rd^ dose) | 01=Yes, 02= No, 88= Don’t know |  |  |
|  |  | IPV | 01=Yes, 02= No, 88= Don’t know |  |  |
|  |  | MR (1^st^ dose) | 01=Yes, 02= No, 88= Don’t know |  |  |
|  |  | MR (2^nd^ dose) | 01=Yes, 02= No, 88 = Don’t know |  |  |
| 1.36 | Did the child receive high dose vitamin A capsule in last 6 months? | | | Yes [1]; No [0]; Don’t know [44]  (skip to ‘1.42’ if ‘0’ or ‘44’) | |
| 1.37 | From where did the child receive the vitamin A dose? | | | Through EPI sessions [1]  Through Vitamin A campaign [2] Others (specify) [3] Don’t know [44] | |
| 1.38 | What did the child consume in last six months after diarrheal episodes? | | | Packed ORS [1]  Homemade ORS [2] Zinc syrup/tablets [3] | |

|  |  | Rice powder [4]  Normal/regular food [5]  Other medicine [6]  NA [99] |
| --- | --- | --- |
|  |  |  |
|  |  |  |
|  |  |  |

**C2. Anthropometry and biochemical measurement**

|  |  |  |  |
| --- | --- | --- | --- |
| Weight (Kg):  If difference > 50gm, do 3rd time | Mother +child:  Mother alone:  Child’s weight: | Mother +child:  Mother alone:  Child’s weight: | Mother +child:  Mother alone:  Child’s weight: |
|  | **Child** | **Mother** | |
| Height (cm):  if difference > 0.5 cm, do 3rd time | 1^st^  2^nd^  3r^d^ | 1^st^  2^nd^  3r^d^ | |
| MUAC (cm):  if difference > 0.2 cm, do 3rd time | 1^st^  2^nd^  3^rd^ | 1^st^  2^nd^  3^rd^ | |
| Haemoglobin concentration of children age between 12-23 months | **.** |  | |
